# Protein Translation and Cell Death: The Role of Rare tRNAs in Biofilm Formation and in Activating Dormant Phage Killer Genes

**DOI:** 10.1371/journal.pone.0002394

**Published:** 2008-06-11

**Authors:** Rodolfo García-Contreras, Xue-Song Zhang, Younghoon Kim, Thomas K. Wood

**Affiliations:** 1 Artie McFerrin Department of Chemical Engineering, Texas A & M University, College Station, Texas, United States of America; 2 Department of Biology, Texas A & M University, College Station, Texas, United States of America; 3 Zachry Department of Civil Engineering, Texas A & M University, College Station, Texas, United States of America; Baylor College of Medicine, United States of America

## Abstract

We discovered previously that the small *Escherichia coli* proteins Hha (hemolysin expression modulating protein) and the adjacent, poorly-characterized YbaJ are important for biofilm formation; however, their roles have been nebulous. Biofilms are intricate communities in which cell signaling often converts single cells into primitive tissues. Here we show that Hha decreases biofilm formation dramatically by repressing the transcription of rare codon tRNAs which serves to inhibit fimbriae production and by repressing to some extent transcription of fimbrial genes *fimA* and *ihfA*. *In vivo* binding studies show Hha binds to the rare codon tRNAs *argU*, *ileX, ileY,* and *proL* and to two prophage clusters D1P12 and CP4-57. Real-time PCR corroborated that Hha represses *argU* and *proL*, and Hha type I fimbriae repression is abolished by the addition of extra copies of *argU*, *ileY*, and *proL*. The repression of transcription of rare codon tRNAs by Hha also leads to cell lysis and biofilm dispersal due to activation of prophage lytic genes *rzpD*, *yfjZ*, *appY*, and *alpA* and due to induction of ClpP/ClpX proteases which activate toxins by degrading antitoxins. YbaJ serves to mediate the toxicity of Hha. Hence, we have identified that a single protein (Hha) can control biofilm formation by limiting fimbriae production as well as by controlling cell death. The mechanism used by Hha is the control of translation via the availability of rare codon tRNAs which reduces fimbriae production and activates prophage lytic genes. Therefore, Hha acts as a toxin in conjunction with co-transcribed YbaJ (TomB) that attenuates Hha toxicity.

## Introduction

Biofilm formation is a complex process that requires the coordinate expression and simultaneous regulation of many genes [1] for reversible and irreversible attachment, microcolony formation, formation of a stable three-dimensional structure, and dispersion [2]. In the absence of conjugation plasmids [3], early steps in biofilm formation require the synthesis of different bacterial surface appendages including flagella that allow reversible attachment [1] and cell motility which is a determinant of biofilm architecture [4]. For irreversible attachment, flagella synthesis is repressed and adhesive organelles like curli fimbriae, encoded by the *csg* operon, and type I fimbriae, encoded by *fim* genes, are required for biofilm formation [1]. The mannose-sensitive, type I fimbriae also mediate adherence that is important for invasion of host cells in some urinary tract infections [5].

In *E. coli,* transcription of the type I fimbriae structural gene cluster *fimAICDFGH* is driven by a single promoter located upstream of *fimA* [6]. The expression of the *fim* operon is altered by phase variation that consists of switching an invertible 314 bp element upstream of *fimA* by two recombinases encoded by *fimB* and *fimE* [7]. *fimB* mediates recombination in both directions (on-to-off and off-to-on) while *fimE* causes on-to-off recombination [8]. Phase variation of the *fim*A promoter as well as transcription of the *fim* genes is influenced by several global, environmental regulators; for example, H-NS reduces the frequency of phase variation [9] and stimulates the transcription of *fimA* in a locked switched-on mutant [10]. The integration host factor IHF encoded by *ihfA* and *ihfB* also plays a dual role in the regulation of type I fimbriae expression since it is required both for inversion of the switching element in both directions and for efficient transcription of *fimA* [11].

In this work, we investigated the mechanism by which Hha (hemolysin expression modulating protein) controls *E. coli* biofilm formation. Hha is a small transcriptional regulator (8 kDa) that belongs to the Hha-YmoA family which includes a group of sequence-related, small proteins involved in gene regulation for Gram-negative bacteria [12]. Mechanistic studies of Hha-mediated gene expression has focused on its repression of the *E. coli* α-hemolysin operon *hly* [12]. However, Hha also represses the expression of many other genes including an endoglucanase of *Clostridium cellulolyticum* [13], and several virulence genes in pathogenic *E. coli* [14], [15] and *Salmonella enterica* [16]. Rather than binding directly to specific DNA sequences in the *hly* operon, Hha exhibits nonspecific DNA binding; therefore, it was proposed Hha binds to H-NS, which binds to specific regions of regulatory sequences in the *hly* operon [17]. We showed previously that *hha* is induced 30-fold in *E. coli* biofilms relative to planktonic cells [18] and that a double deletion of *ybaJ-hha* affects biofilm formation, motility, and plasmid conjugation [19]. Here we demonstrate that Hha represses biofilm formation by repressing type I fimbriae formation. We also present evidence of a novel, translation-based mechanism for the repression of fimbrial proteins: Hha represses minor regulatory tRNAs that participate in the modulation of the expression of the recombinases FimB and FimE and the structural fimbrial proteins (FimAICDFGH). In addition, Hha exerts a toxic bacteriolytic effect through repression of the rare tRNA; by altering protein translation with the rare tRNAs, general proteases are induced (e.g., ClpP/ClpX) as well as lytic cryptic phage genes. The toxic effects of Hha are counteracted by YbaJ.

## Results

### Hha represses initial biofilm formation and inhibits cell growth

**Figure 1:**
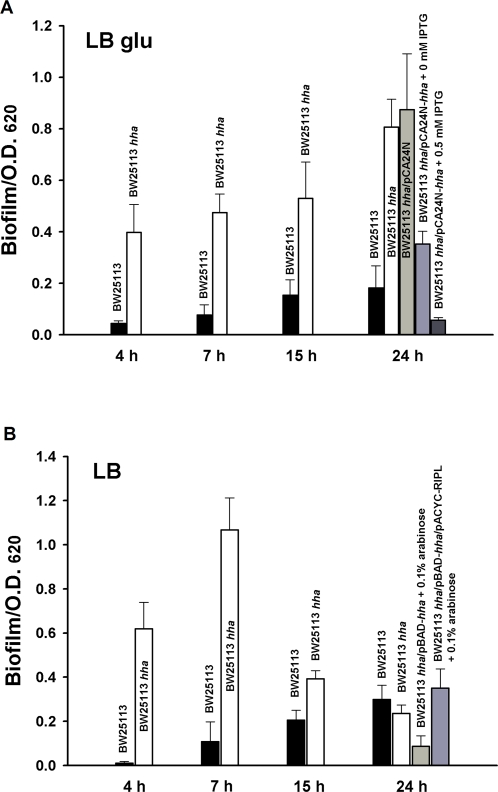
Hha decreases total biofilm formation. Biofilm formation normalized by cell growth shown after 4 h, 7 h, 15 h, and 24 h at 37°C for the wild-type BW25113 and BW25113 *hha* in (A) LB glu and (B) LB medium. Complementation of biofilm formation is indicated in LB glu using pCA24N-*hha* (A), and in LB using pACYC-RIPL to express rare codons (B). Each experiment was performed in triplicate, and one standard deviation is shown.

Since biofilms have a complex time- and temperature-dependent formation process [20], we analyzed biofilm formation of the *hha* deletion mutant as a function of time, both in LB and LB glucose (LB glu), since Hha has been linked to catabolite repression [21]. In LB glu, the isogenic *hha* deletion mutant formed an order of magnitude more biofilm initially on a per cell basis than the wild-type strain, and Hha was the cause of this change in biofilm formation since expressing *hha* in trans reduced biofilm formation to levels even lower than wild type (Figure 1A). Similarly, in LB, the *hha* deletion increased biofilm formation 58-fold (at 4 h) but this difference diminished with time (Figure 1B). As in LB glu, complete inhibition of biofilm formation was observed at 4 h in the complemented *hha* mutant in LB medium with 2 mM IPTG; complementation also occurred in both media at 0.05 mM concentrations of IPTG (data not shown). Hence, Hha inhibits biofilm formation dramatically and consistently. Note the *hha* deletion mutation did not affect the growth of planktonic cells in both LB and LB glu media (specific growth rates unchanged).

**Figure 2:**
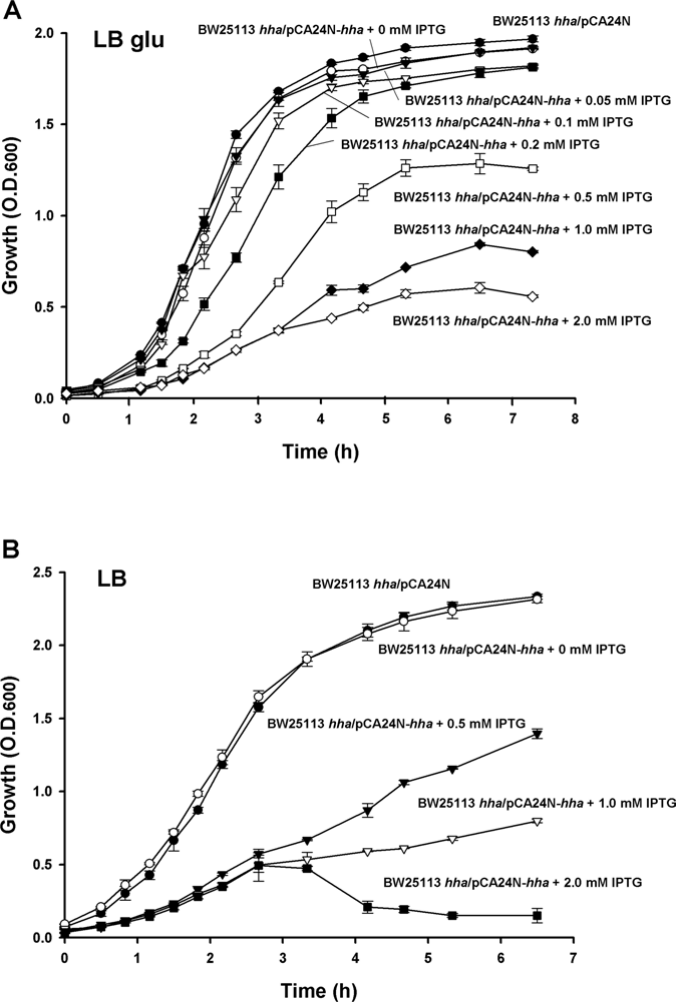
Hha reduces cell growth. Growth of BW25113 *hha*/pCA24N-*hha* at 37°C in (A) LB and (B) LB glu. The experiment was performed in triplicate, and one standard deviation is shown.

While overexpressing Hha to complement biofilm formation, we noticed it decreased cell growth in both media as a function of expression level (Figure 2); hence, overexpression of Hha is toxic. It was verified that the toxic effect observed was a consequence of Hha overexpression and not an artifact created by the addition of IPTG since growth of planktonic BW25113 *hha*/pCA24N in both LB and LB glu was not significantly decreased by the addition of IPTG (2 mM) (data not shown).

### Hha inhibits biofilm formation by repressing type I fimbriae

To elucidate the mechanism by which Hha inhibits biofilm formation, a series of DNA microarrays for biofilm cells cultured in glass wool were performed (4 h in LB and a temporal series of 4 h, 15 h, and 24 h in LB glu for a *hha* mutant vs. wild-type) since Hha affects the production of multiple proteins and causes pleiotropic effects on catabolite repression and other systems [21]. The biofilm mass formed by the *hha* mutant in the glass wool cultures was significantly higher than for the wild-type strain which is consistent with the biofilm quantification experiments using crystal violet staining in 96-wells polystyrene plates. Deleting *hha* impacted the differential expression of a large number of genes (1.3 to 5.5% of the *E. coli* genome).

In order to simplify the analysis, we focused on those genes that appeared differentially regulated in at least two of the four microarrays (Table S1 and S2). Among the induced genes, stress response genes were well represented as expected [18], [20], [22], [23] including those related to osmolarity (e.g., *osmCEY*, *ybaY,* and *ygaM*) which reflects the role of Hha as an osmoregulator [24]. Additionally, four regulators were induced including the biofilm regulator *bssR* that regulates biofilm by influencing the uptake and processing of autoinducer-2 (AI-2) as well as indole transport [25] and the carbon storage regulator *csrA* that regulates the cell central carbon flux, represses biofilm, and activates biofilm dispersal [26]. Also induced were the putative transcriptional regulator *yiaG*, induced by AI-2-mediator MqsR [27], and *ihfA* (*himA*), which together with *ihfB* (*himD*), constitutes the global regulator IHF that is required for site-specific recombination, DNA replication, transcriptional control, and fimbriation [28]. *fimA,* the major constituent of type I fimbriae and an early stage biofilm factor, was also induced upon deletion of *hha* in early biofilms (4 h) in both LB and LB glu (Table S1). The induction of *fimA, ihfA,* and *ihfB* upon deleting *hha* gave us the first clues that part of the mechanism of Hha inhibition of biofilms is repression of type I fimbriae. Interestingly, *ybaJ* was significantly induced in all of the microarrays. Since this gene is part of an operon with *hha* and *ybaJ* is the first gene downstream of their common promoter, the microarray data indicate that Hha represses *ybaJ* as well as its own transcription, a common property among the histone-like regulatory proteins like IHF [29], H-NS [30], and HU [31].

The repressed genes upon *hha* deletion included those related to metabolism, genes with unknown function, the tRNA *proM,* and *fimD,* a constituent of the type I fimbriae chaperone usher pathway. *fimD* repression was another link between Hha and type I fimbriae.

*tnaA* that encodes tryptophanase, which synthesizes the cell signal indole that is an inhibitor of *E. coli* biofilm formation [32], [33], was induced 2.8-fold at 4 h and then repressed 5-fold at 15 h (LB glu arrays). This significant repression of *tnaA* at 15 h should decrease indole synthesis and contribute to enhanced biofilm formation for the *hha* mutant. Corroborating this whole transcriptome data, the extracellular indole concentration of the *hha* mutant was 12±2-fold lower at 15 h in LB glu (435±65 µM for the wild-type strain and 25±3 µM for the *hha* mutant) which is consistent with the microarray data.

### Confirmation that Hha represses Type I fimbriae via yeast agglutination

**Figure 3:**
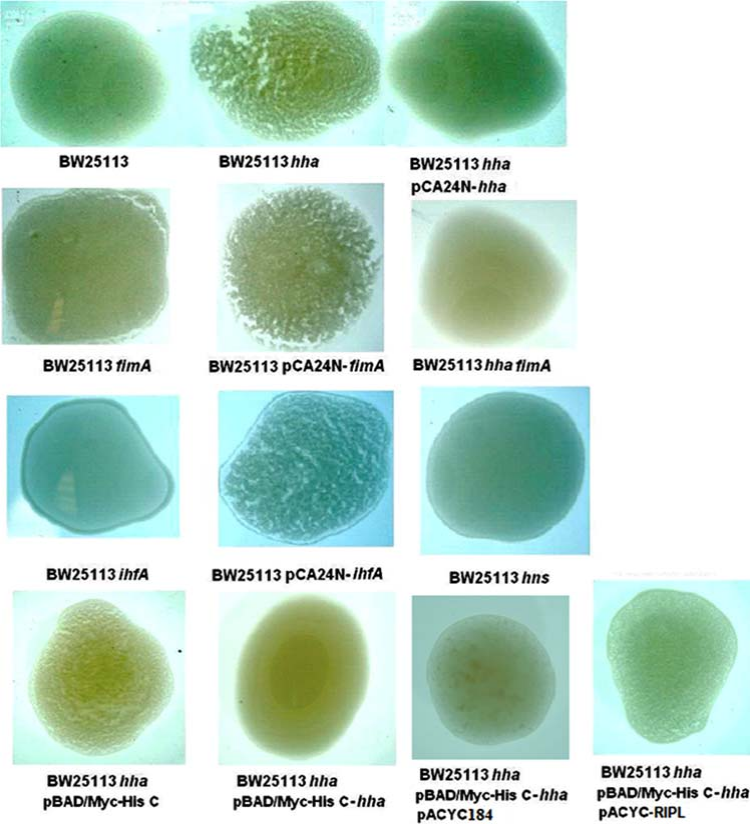
Hha decreases Type I fimbriae formation. Type I fimbriae were assayed via the yeast agglutination assay with various BW25113 mutants and complemented strains. pACYC-RIPL encodes extra copies of the rare codon tRNAs *ileY*, *argU*, *leuW* and *proL*, and pACYC184 is the empty vector control. The experiments were performed in triplicate.

To confirm the DNA microarray prediction that Hha represses fimbriae production, a direct evaluation of type I fimbriae production in the *hha* strain was performed using a classical yeast agglutination assay on glass slides [34] (Figure 3). The results confirm our hypothesis since the *hha* deletion mutant showed much higher yeast agglutination than the wild-type strain (fimbriae increase agglutination), and the enhanced agglutination was completely abolished by the overexpression of Hha from both pCA24N-*hha* and pBAD-*hha.* These results corroborate the DNA microarray data and show that Hha represses the production of type I fimbriae. As expected, the isogenic *fimA* and *ihfA* deletion mutants did not show agglutination, and the agglutination phenotype was recovered by the complementation with pCA24N-*fimA* and pCA24N-*ihfA*, respectively (Figure 3).

All cases of positive agglutination were blocked by the addition of mannose 2% which shows that the observed yeast agglutination was indeed caused by the expression of mannose-sensitive type I fimbriae [34]. Additionally, the yeast agglutination phenotype of a *hns* isogenic mutant was measured since H-NS binds Hha making a heteromeric complex that regulates gene expression [17], and H-NS decreases the frequency of *fimA* promoter switching [9] as well as activates transcription from the *fimA* promoter in a locked-on mutant [10]. The results corroborated that H-NS is an activator of *fimA* expression [10] since no agglutination was observed for the *hns* deletion mutant under the assayed conditions (Figure 3); therefore, Hha and H-NS play opposite roles in the regulation of type I fimbriae expression.

### Hha partially represses biofilm formation through type I fimbriation via *fimA* and *ihfA*

Along with *fimA,* the main structural component of type I fimbriae, *hha* deletion induced *ihfA*; *ihfA* is a subunit of the IHF global regulator that is required for the normal expression of *fimA*, as mutations in *ihfA* or in the IHF beta-subunit *ihfB* (also induced upon *hha* deletion) prevent the phase variation expression of *fimA* [11]. Furthermore, when the *fim* switch, containing the *fimA* promoter region, is locked in the on phase, lesions in either *ihfA* or *ihfB* result in a sevenfold reduction in expression of *fimA* [11]. Therefore, since the overall effect of IHF in *fimA* regulation and fimbriae expression is positive, and since both *ihfA* and *fimA* are induced upon *hha* deletion, we hypothesized that another part of the mechanism by which Hha represses biofilm formation is through the diminution of type I fimbriae through the repression of *fimA*, either directly or indirectly through the repression of IHF.

To test this hypothesis, we evaluated biofilm formation of isogenic *fimA* and *ihfA* mutants and found the *fimA* deletion completely abolished biofilm formation in LB media at 7 h and 24 h and reduced biofilm formation relative to the wild-type strain by 70±2% in LB glu at 24 h (data not shown); these results were expected given the known role of type I fimbriae in biofilm production [35]. Moreover, the *ihfA* mutant had identical biofilm formation as the *fimA* mutant in both LB and LB glu media since it also completely abolished biofilm formation at 7 and 24 h in LB and repressed by 71±2% biofilm formation in LB glu at 24 h (data not shown). This is the first report of the role of IHF in enhancing biofilm formation.

Since the biofilm data were consistent with our hypothesis, biofilm formation of the *hha/fimA* and *hha/ihfA* double mutants was evaluated since if induction of *fimA* via *ihfA* is important for the increase in biofilm upon *hha* deletion, then *hha* deletion in a *fimA* or *ihfA* background should not increase biofilm formation to the same extent as the *hha* deletion in a wild-type background because the production of type I fimbriae should be blocked by the second deletion (*fimA* or *ihfA*). As a control, we tested biofilm formation of the double mutants *tqsA fimA* and *bssS fimA* since the *bssS* and *tqsA* deletions significantly increase biofilm formation in LB glu [25], [36], and the mechanism for this increase is not related to the induction of type I fimbriae but instead involves a decrease in indole for the *bssS* mutant [25] and deficient transport of AI-2 for the *tqsA* mutant [36]. Hence deleting *fimA* in these backgrounds should not inhibit the biofilm formation to the same extent as that of a wild-type strain. Corroborating our hypothesis, biofilm formation of the *hha fimA* and *hha ihfA* double mutants was completely inhibited in LB at 24 h and 50% inhibited in LB glu at 24 h (showing a similar effect as *fimA* and *ihfA* single mutants) (data not shown). In contrast, the *bssS fimA* and *tqsA fimA* double mutants still produced a significant amount of biofilm (data not shown). Therefore, Hha represses biofilm formation through type I fimbriae production.

### Hha represses transcription of *fimA, ihfA*, and *ybaJ* but does not change *fimA* promoter switching frequency

**Figure 4:**
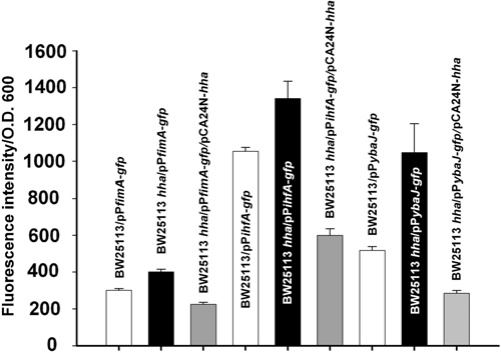
Hha inhibits transcription of *fimA*, *ihfA*, and *ybaJ*. Transcription rates were quantified using GFP fluorescence intensity as normalized by cell turbidity at 600 nm via pP*fimA*-*gfp*, pP*ihfA*-*gfp,* and pP*ybaJ*-*gfp*. The experiments were performed at least 2 times, and one standard deviation is shown.

In order to further validate the hypothesis of Hha biofilm inhibition by type I fimbriae diminution through the repression of *ihfA* and *fimA*, transcriptional activity of the *fimA* and *ihfA* promoters were evaluated in the wild-type strain, the *hha* mutant, and upon overexpression of Hha from pCA24N-*hha*. These two promoters as well as the *ybaJ*-*hha* promoter (in order to test if Hha repressed its own and *ybaJ* transcription) were cloned into pPROBE-*gfp*[tagless] that contains a promoterless, short-lived GFP [37]. As expected, the fluorescence signal derived from the transcriptional activity of the three promoters (*fimA*, *ihfA*, and *ybaJ*-*hha*) was higher in the *hha* mutant than in the wild-type strains (Figure 4). Also, for all three promoters, the fluorescence was significantly reduced by the overexpression of Hha from pCA24N-*hha* upon the addition of IPTG 0.5 mM (Figure 4). Moreover, repression of *fimA, ihfA*, and *ybaJ* by Hha was further corroborated by quantitative real time PCR (RT-PCR) using RNA from 15 h LB glu biofilm cells since we found *fimA, ihfA*, and *ybaJ* were induced in the *hha* mutant as expected: *fimA* 6.1±0.2-fold, *ihfA* 3.1±0.1-fold, and *ybaJ* 8.0±0.5-fold. These results corroborate that the activities of the three promoters are repressed either directly or indirectly by Hha.

Since Hha repression of *fimA* transcription can be a consequence of shifting the *fimA* promoter orientation to the off position or a result of repression of *fimA* transcription, or a simultaneous change in both factors (promoter orientation and transcription), we compared the *fimA* promoter orientation in the wild-type strain and in the *hha* mutant by PCR-restriction enzyme analysis [38]. No appreciable change was found in the promoter orientation, which was mainly in the on position for both strains (data not shown); hence, Hha represses type I fimbriae transcription but does not affect the *fimA* promoter orientation significantly.

### Hha binds prophage genes and rare tRNA regions *in vivo*

To probe the whole genome to find which promoters Hha represses and to avoid its nonspecific binding *in vitro* [17], we performed a nickel-enrichment DNA microarray using biofilm cells. In this assay, formaldehyde is used to cross-link His-tagged Hha to the DNA fragments to which Hha is bound. Then the Hha:DNA cross-linked complexes are purified by a His-tag nickel affinity chromatography and identified using a DNA microarray [39]. Note that H-NS and any other Hha-binding partner were present in the cells during this experiment and thus the identified targets should be physiologically-relevant. For the nickel enrichment DNA microarray assays, biofilm cells were cultured for 24 h at 37°C in LB glu medium, and under these conditions the *hha* mutant makes 3-fold more biofilm than the wild-type.

The nickel-enrichment DNA microarrays identified 33 genes and 37 intergenic regions enriched at least 4-fold in the *hha* mutant upon the induction of Hha in trans (Table S3). The intergenic regions and the promoters of the genes enriched represent possible *in vivo* DNA binding sites for Hha. Among the putative Hha DNA binding sites identified, 15 genes or intergenic regions belonging to the DLP12 and CP4-57 prophage gene clusters were found as well as regions close to *argU* (encodes an arginine tRNA that recognizes a rare codon), *ileY* (encodes an isoleucine tRNA that recognizes a rare codon), and *rzpD* (encodes a predicted murein endopeptidase of DLP12 prophage); these regions are related to Hha toxicity (below). In addition, other putative Hha binding sites were identified such as those for fimbrial-like adhesins, acid resistance, and virulence genes (which supports the role of Hha as a modulator of the expression of virulence factors). We confirmed that Hha regulates the expression of three of the identified genes, *ybcW*, *ypjC*, and *yeeO*, that were enriched 9.2-, 4.6-, and 147-fold, respectively (Table S3), by RT-PCR; using biofilm cells grown as in the nickel-enrichment assay, *ybcW*, *ypjC*, and *yeeO* were induced by 17±2, 9±1, and 50±5-fold, respectively. These data confirm Hha binds these chromosomal regions and induces their transcription. Another set of Hha nickel-enriched microarrays performed in *E. coli* BL21 (DE3) by us confirmed that Hha binds to the rare tRNA regions that were identified in BW25113 (*argU* and *ileY*), to another two rare codon tRNAs (*ileX* and *proL*), and to the *hha* neighbor gene *ybaJ* (data not shown). To determine if H-NS influences Hha-DNA interactions, we performed the same experiment in an isogenic *hha/hns* double mutant and found that the number of putative Hha binding sites decreased to 19 genes and 25 intergenic regions with 26 of these targets common between the two backgrounds and 18 were specific for the *hha*/*hns* mutant. Therefore, 44 Hha-DNA interactions were lost in the absence of H-NS (Table S3), showing that H-NS confers some DNA binding specificity to Hha by forming heteromeric complexes.

### Hha represses the transcription of rare codon tRNAs (RT-PCR)

Given that the nickel-enrichment DNA microarray analysis shows Hha binds near *argU* which controls gene regulation through rare arginine codons in *S. typhimurium* [40] as well as binds *ileX, ileY,* and *proL* which encode rare codons for *E. coli*, we hypothesized that Hha inhibits type I fimbriae through the repression of rare codon tRNAs that leads to a diminution of the translation rate of fimbrial proteins. To test this hypothesis, RT-PCR was conducted for *argU* and *proL* tRNAs in exponentially-growing planktonic cells upon *hha* overexpression from pCA24N-*hha*. As expected, the concentrations of *argU* and *proL* tRNA in the wild-type strain were higher than in the *hha* mutant harboring pCA24N-*hha* (3.2±0.1-fold for *argU* and 4.1±0.2-fold for *proL*), and the difference increased upon inducing *hha* by the addition of 0.5 mM of IPTG (5.6±0.2-fold for *argU* and 8.2±0.4-fold for *proL*). In contrast, Hha induction did not affect the expression of the abundant tRNAs *ileV*, *leuP*, and *argQ*. Hence, Hha specifically represses the transcription of *argU* and *proL* rare tRNAs.

### *E. coli* type I *fimbriae* genes are biased toward rare codons

Since Hha binds and represses the transcription of rare codon tRNA genes, the abundance of rare codons were analyzed in the type I fimbriae regulators *fimB* and *fimE,* in *fimZ* (not known to regulate type I fimbriae in *E. coli* but it is a fimbriae regulator in *Salmonella* sp.), and in the type I fimbrial structural gene cluster *fimAICDFGH*. The rare codons studied were the arginine AGG and AGA codons recognized by *argU*, the isoleucine ATA codon recognized by *ileX* and *ileY*, and the proline CCC codon recognized by *proL* since Hha binds to these three RNA loci *in vivo*. Additionally, the abundance of the rare leucine TTG and CTA codons, recognized by *leuX* and *leuW*, respectively, and the rare threonine ACA codon, recognized by *thrU* were also examined.

**Figure 5:**
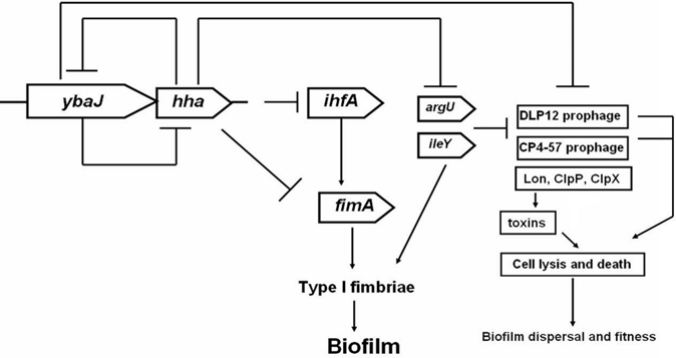
Model for Hha regulation of *E. coli* biofilm and cell death. → indicates induction, and ⊥ indicates repression.

The sequence analysis showed that the rare arginine AGA codon was present in all the studied genes at least one time and in all cases the frequency was higher than expected according the *E. coli* codon usage for the complete genome (Table S4). Also, the rare arginine AGG codon was present at a higher frequency than expected in *fimZ*, *fimI*, *fimC*, *fimF,* and *fimG* (Table S4). The rare isoleucine ATA codon was also present at a higher frequency than expected in all the genes except *fimH* and *fimA* and was especially abundant in *fimZ* and *fimB* having six ATA codons each. The rare proline CCC codon was present at a frequency higher than the expected in *fimZ*, *fimB*, *fimC*, *fimD*, and *fimF* and was especially abundant in *fimD* which contained seven CCC codons. Moreover, the rare leucine TTG codon was present at a frequency higher than expected in all genes except *fimA, fimE*, and *fimD*, the rare leucine CTA codon was present in *fimZ*, *fimB*, *fimI*, *fimF*, and *fimG*, and the rare threonine ACA was present in all genes but *fimE*, *fimI*, *fimF,* and *fimH*. These results are summarized in Table S4, and a scheme summarizing the regulatory effects of Hha in biofilms, and its role in cell death is presented in Figure 5. Taken together, these results show that a rare codon usage is a characteristic of the regulatory and structural type I fimbriae genes.

### Hha represses fimbriae production by repressing rare codons (agglutination)

To show directly that Hha works by repressing the synthesis of rare codons, we added extra copies of rare tRNAs through plasmid pACYC-RIPL during Hha overexpression (from pBAD-*hha*) during the yeast agglutination assay. The extra copies of rare tRNA counteracted Hha mediated inhibition of yeast agglutination which corroborates that part of the Hha type I fimbriae inhibition mechanism is through the repression of rare coding tRNAs (Figure 3). Note there was no interference of Hha-mediated inhibition of yeast agglutination with the empty control plasmid pACYC184 (Figure 3).

### Hha toxicity and biofilm formation are attenuated by adding rare codon tRNAs

**Figure 6:**
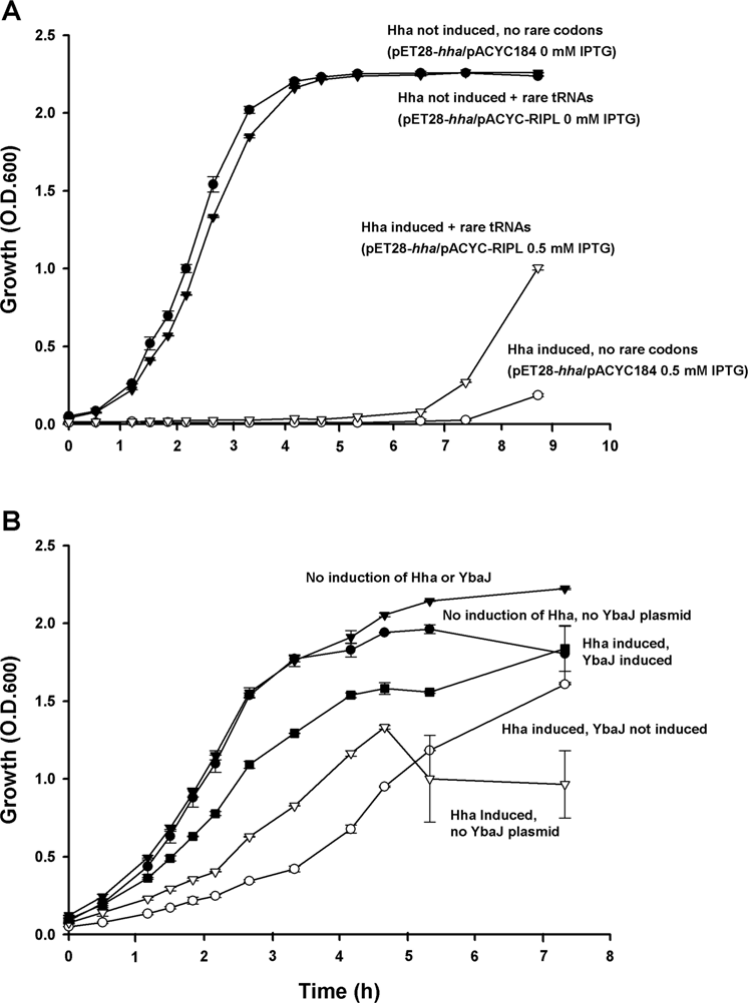
Extra copies of rare codon tRNAs and YbaJ reduce Hha toxicity. Inhibition of cell growth in LB at 37°C by expressing Hha from plasmid pET28-*hha* with 0.5 mM IPTG in *E. coli* BL21 (DE3)/pACYC184 (empty vector control) or in BL21 (DE3)/pACYC-RIPL cells expressing extra copies of the rare codon tRNAs *ileY*, *argU*, *leuW* and *proL* (A). Effect of YbaJ overexpression from pBAD-*ybaJ* in *E. coli* K-12 BW25113 *hha* for Hha-mediated toxicity from pVLT31-*hha* in LB at 37°C; Hha was induced by the addition of 2 mM IPTG and YbaJ was induced by the addition of 0.1% *L*-arabinose (B). The experiments were performed in duplicate, and one standard deviation is shown.

Given that Hha is toxic (Figure 2) and represses the transcription of *argU* and *proL,* we hypothesized that part of the toxicity mechanism may involve depletion of rare codon tRNAs since the diminution of specific rare codon tRNAs by minigenes is toxic with the rare arginine AGG and AGA codons (*argU*) and the rare isoleucine ATA codon (*ileX* and *ileY*) most toxic [41]. In order to test this hypothesis, overexpression of Hha was assayed with extra copies of *ileY*, *argU*, *leuW,* and *proL* using pACYC-RIPL; the addition of additional copies of rare codon tRNAs through pACYC-RIPL significantly decreased Hha toxicity (Figure 6A). Furthermore, the addition of additional copies of rare codon tRNAs through pACYC-RIPL significantly increased biofilm formation of BW25113 *hha*/pBAD-*hha* (0.35±0.09 vs. 0.09±0.05) (Figure 1B). Hence Hha toxicity, fimbriae production, and biofilm formation are linked to the rare codons.

### YbaJ attenuates Hha toxicity

Based in the following observations, our data suggest that Hha and YbaJ may form a toxin-antitoxin module with Hha the toxin (Figure 2) and the poorly-characterized YbaJ the antitoxin: (i) *hha* and *ybaJ* are in the same operon, (ii) the separation between the genes is short (25 bp), (iii) both proteins are small, (iv) Hha exerts negative transcriptional self-regulation, and (v) overexpression of Hha is toxic; all of these are characteristics of a toxin-antitoxin pair [42]. Further supporting this hypothesis is that the *ybaJ-hha* operon is highly induced in *E. coli* biofilms [18], and toxin-antitoxin modules arise at a higher frequency in stationary growth phase and in biofilms in association with persister cells [43].

In order to test if YbaJ counteracts the toxic effects of Hha, we assayed the effect of YbaJ overexpression in Hha toxicity using a two plasmid system consisting of pBAD-*ybaJ,* to induce YbaJ by the addition of 0.1% arabinose, and pVLT31-*hha*, to induce Hha by the addition of 2 mM IPTG. As expected, the induction of Hha without YbaJ was toxic to the cell, and the toxicity of Hha was attenuated by simultaneous YbaJ overexpression (Figure 6B). Hence, YbaJ expression diminished Hha toxicity.

### Hha causes cell lysis by inducing prophage lytic genes and by activating toxins via proteases

Given that Hha binds *argU* and *ileY in vivo* (Table S3) and that Hha toxicity in part is mediated by depletion of rare codon tRNAs (Figure 6A), the gene context of these tRNAs was examined. Two of the tRNAs that Hha binds, *argU* and *ileY,* are close to prophage gene clusters since 37 DLPD-12 prophage genes are located immediately downstream to *argU*. We have found 10 of these DLPD-12 prophage genes are differentially-regulated in biofilms relative to planktonic cells [23], [36]. Furthermore, 26 CP4-57 prophage genes are located five genes upstream *ileY*, and we have found eight of these genes are differentially regulated in biofilms [23], [36]. Of these 18 prophage genes related to biofilms, 10 are significantly repressed in *hha* mutant biofilms at 15 h and 24 h in LB glu (Table S6), and no DLPD-12 or CP4-57 prophage gene is induced in the absence of Hha at any time in any condition; therefore, it appears Hha either directly or indirectly activates the expression of some phage genes that cause cell lysis (probably by repression of *argU* and *ileY*). Specifically, upon *hha* deletion, prophage gene *essD* is repressed 2.6-fold at 24 h in LB glu; EssD encodes a putative holin [44] and forms an operon with *ybcS* and *rzpD* (http://ecocyc.org/) that encode a putative endolysin [44] and a predicted murein endopeptidase (http://ecocyc.org/) respectively. *alpA* is also repressed 4.9-fold at 15 h in LB glu and encodes a transcriptional activator of a P4-like cryptic prophage [45]. Furthermore, *yfjZ-ypjF* is repressed 2.4-fold at 24 h in LB glu and 6-fold at 15 h LB glu, respectively, and encodes a toxin-antitoxin module [46]. Corroborating the transcriptome microarray data and the nickel-enrichment DNA microarray data, induction of Hha in the *rzpD*, *yfjZ*, *alpA,* and *appY* deletion mutants resulted in significantly less growth inhibition than in the wild-type *hha* mutant background (Figure S1) which indicates that these DLP12 and CP4-57 prophage genes are involved in Hha toxicity. In contrast, Hha overexpression did not have a significantly different effect on the growth of the other prophage mutants like *ybcY*, *ybcN*, *ypjF*, *yfjF*, *yfjG*, *ybcS*, *essD*, and *ompT* (data not shown).

To investigate further the hypothesis that Hha overexpression activates lytic prophage genes leading to cell death, formation of lysis plaques in soft agar upon Hha overexpression from pCA24N-*hha* by the addition of 1 mM IPTG was assayed; surprisingly, we found that Hha overexpression causes the formation of cell plaques (Figure S2) and that the treatment of BW25113 wild type with the lysate extracted from those plaques does not cause lysis by itself. This data agree with the lysis seen in Figure 2B upon induction of Hha with 2 mM IPTG. Hence, Hha toxicity leads to cell lysis and may not involve the production of active phage, however we can not rule out this possibility.

To explore further the toxicity mechanism of Hha, a DNA microarray was performed for Hha overexpression using BW25113/pCA24N-*hha* suspension cells grown at 37°C with 2 mM IPTG to induce *hha*. Hha induced 39 genes and repressed 5 genes more than 4-fold (Tables S7A and S7B). As expected, *hha* was induced 6.5-fold, a level of induction comparable to the 30-fold induction of *hha* in biofilms [18]; hence, the induction levels of *hha* in our experiments are not far from those naturally observed and the effects of Hha overexpression then are physiologically relevant. Of those induced genes, three encoded the proteases ClpP, ClpX and Lon which degrade many proteins including the antitoxins of several toxin-antitoxin pairs [47]; therefore, it is likely that induction of these proteases by Hha degrades antitoxins leaving the toxins free to exert inhibitory effects on growth. Supporting our hypothesis, overexpressing Hha in both *clpX* and *clpP* deletion mutants resulted in significantly less growth inhibition than in the wild-type *hha* mutant background (Figure S1E). Moreover, three antitoxin genes were also induced: *dinJ*, *relB* and *yefM*, which may be a response of the cell to compensate for the low antitoxin protein levels, and RelB and YefM are known targets of Lon [47]. In addition, 12 heat shock proteins (mainly chaperones) were induced upon Hha overexpression which is reasonable in that Lon degrades RelB which stimulates *relBE* transcription [48] causing RelE to accumulate in excess which causes translation inhibition and perhaps the accumulation of misfolded proteins that trigger the activation of the heat shock chaperones.

The Hha overexpression microarray data were validated by RT-PCR since RT-PCR showed *lon*, *dinJ,* and *yefM* were induced 2.5±0.4-fold, 4.6±0.4-fold, and 3.3±0.3-fold, respectively. Additionally, induction of Hha in the *relE* and *yoeB* toxin deletion mutants resulted in significantly less toxicity than in the *hha* mutant (data not shown) corroborating that Hha expression induces other toxins and that these toxins are required for Hha toxicity. Taken together, these results suggest that an important part of Hha toxicity may be indirect activation of several toxins of toxin-antitoxin pairs via the degradation of their antitoxins by regulatory proteases.

### YbaJ DNA-binding regions

To research the role of YbaJ in the attenuation of Hha toxicity, nickel enriched DNA microarrays were also performed using pCA24N-*ybaJ* to identify putative *in vivo* binding sites for YbaJ in biofilms cells. The results indicate that YbaJ may bind to 21 genes and two intergenic regions (Table S3). Of these putative targets, the more interesting include the *ybaJ* gene itself, indicating that YbaJ may regulate *hha* and its own expression. Also, YbaJ binds *sfmH,* which encodes a predicted *Salmonella* fimbrial-like adhesin protein that is only one gene away from *fimZ* and *argU* (Hha binds these sites), which indicates Hha and YbaJ bind nearby loci and may compete for the regulation of genes near the binding sites. Since Hha exerts a toxic effect counteracted by YbaJ, YbaJ binding *ybcS*, which encodes the prophage endolysin gene of the DLP12 prophage cluster genes that lies near *argU* and which is repressed in *hha* mutant biofilms, indicates YbaJ may repress this gene as part of its mechanism. Additionally, YbaJ binds *ariR* (*ymgB*), which encodes an acid resistance gene involved in the regulation of biofilm formation and which we found to be a structural Hha analog [39].

### Hha induces biofilm dispersal

Since bacterial cell death produced by bacteriophage [49] and the activation of killing proteins [50] promotes biofilm dispersal in pseudomonads, we explored the possibility that Hha could cause dispersal in biofilms. To test this hypothesis, biofilm cells of *E. coli* K12 BW25113/pCA24N-*hha*/pCM18 were cultured in LB glu at 37°C in a continuous flow cell system for 42 h to form a biofilm, then 1 mM IPTG was added to induce Hha expression for 6 h. We found that Hha expression reduced significantly the biomass, substratum coverage, and average thickness by 28±20%, 38±20%, and 27±16%, respectively, compared to the biofilm before IPTG addition. In contrast, when IPTG was not added, biomass, substratum coverage, and average thickness increased by 32±20%, 32±19%, and 28±18%, respectively at 48 h. Therefore, Hha inhibits biofilm formation and contributes to the detachment of preexisting biofilms.

## Discussion

Since Hha was discovered as a repressor of episomal hemolysin production [51], it has been studied extensively to discern its regulatory role in the cell. Hha affects the production of multiple proteins and causes pleiotropic effects for genes related to catabolite repression [21]; Hha also affects other phenotypes including plasmid supercoiling [52] and insertion sequence transposition [53]. Hha is conserved in pathogens [53] and also represses the expression of other virulence genes in pathogenic *E. coli* including episomal Vir adhesion [14] and the locus of enterocyte effacement [15].

Here we investigated the relationship between Hha and biofilm formation in *E. coli* K12 in an attempt to explore its role in cellular physiology, a role that remains unclear despite considerable investigation [12]. Previously we found that the *ybaJ*-*hha* operon was induced significantly in *E. coli* biofilms [18] and that Hha and YbaJ regulate biofilm formation in cells harboring the derepressed conjugative plasmid R1drd19 [19]. Through our temporal study, we show here that Hha controls many genes in both LB and in LB glu including the main structural subunits of the cellular appendage required for attachment, type I fimbriae (*fimA*). This implies Hha is important for early steps in biofilm formation. In agreement with this, the *hha* deletion had the most effect in LB at early times (Figure 1B). Other lines of evidence found here linking Hha to fimbriae include (ii) *ihfA* encoding a subunit of the IHF regulator that is an activator of type I fimbriae production via the *fimA* promoter [11] is activated in all the LB glu microarrays upon *hha* deletion (Table S1), (iii) Hha represses *fimA* and *ihfA* transcription in biofilm cells as shown by RT-PCR, (iv) *fimA* and *ihfA* mutations abolish biofilm formation, (v) deleting *hha* along with either *fimA* and *ihfA* (double mutants) fails to increase biofilm formation, (vi) transcription of *fimA* and *ihfA* is induced in the *hha* deletion mutant and is inhibited by overexpressing *hha* (GFP reporter constructs) (Figure 4), and (vii) type I fimbriae production dramatically increases in the absence of *hha* and Hha overexpression inhibits type I fimbriation (Figure 3). Together these results show Hha represses fimbriae synthesis although it does not affect *fimA* promoter orientation.

Although the regular DNA microarrays give valuable information about how Hha controls *E. coli* biofilm formation (Tables S1, S2, and S6), it is not possible to determine which effects were mediated directly by Hha transcriptional control. However, an intrinsic difficulty for the identification of the promoters which are regulated directly by Hha *in vivo* is that *in vitro* approaches like electrophoretic mobility shift assays are not useful for the identification of Hha binding sites since Hha forms heteromeric complexes with other proteins like H-NS that exhibit different DNA binding properties, and Hha alone exhibits nonspecific DNA binding [17]. Therefore, we utilized an *in vivo* nickel enrichment DNA microarray assay to identify DNA binding based on methods such as nickel agarose-based chromatin enrichment [54]. We found that *in vivo,* Hha binds its own promoter; this is consistent with induction of *ybaJ* in all the regular *hha* DNA microarrays (corroborated with RT-PCR in biofilm cells) and with the enhancement of transcription from the *ybaJ-hha* promoter observed in the *hha* mutant (Figure 4). These experiments demonstrate that Hha regulates its own transcription via negative feedback.

Furthermore, the nickel enrichment DNA microarrays show Hha binds *in vivo* to the rare tRNA loci *argU*, *ileX, ileY,* and *proL* as it reduces fimbriae production. *Salmonella* possesses a fimbrial cluster whose regulation is different than that in *E. coli* since in *Salmonella* there is no phase variation control by promoter switching. The *Salmonella* cluster is composed of *fimZYW* and *fimU*, and *fimU* is a tRNA homolog of *argU* [55], [56]. *fimU* tRNA has an important role in the regulation of type I fimbriae in *Salmonella* since rare arginine AGA codons are prevalent in the three *fim* regulatory genes (*fimZYW*), and production of FimY and type I fimbriae are reduced in the absence of *fimU* since the *fimU* mutation results in the inhibition of efficient FimY translation [57]. Other examples of the regulatory effects of tRNA on fimbriae are that the leucine tRNA *leuX* that recognizes the rare leucine codon TTG controls the expression of the *fimB* recombinase in uropathogenic *E. coli* [58], and *argU* controls the expression of *fimY* in *Salmonella*. Both regulatory tRNAs exert their effect by the attenuation of the translational rate of FimB and FimY since these genes are biased for *argU* and *leuX* codons [57], [58].

The repression of the *argU*, *ileX, ileY,* and *proL* rare tRNAs by Hha should be physiologically relevant for the translation of proteins with a high proportion of rare AGG, AGA, ATA, and CCC rare codons, respectively. Our sequence analysis of *fimZ*, *fimB, fimE,* and the type I fimbrial gene cluster *fimAICDFGH* show all the genes contain an abundance of rare codons recognized by *argU*, *ileX, ileY*, *proL*, as well as rare threonine and leucine codons recognized by *thrU* and *leuXW,* respectively (Table S4). Given that both *fimE-* and *fimB*-promoted switching are stimulated by the amino acids alanine, isoleucine, leucine, and valine [59] and that the use of minor codons is a general mechanism to control the temporal expression of specialized genes at the post-transcriptional level [60], we propose that Hha inhibits the production of type I fimbriae not only by the transcriptional inhibition of *ihfA* and *fimA* but also by the diminution of the availability of *argU*, *ileX, ileY*, and *proL* tRNAs and this leads to a reduction in the translation of the type I fimbriae proteins. This hypothesis was corroborated since the addition of extra copies of the genes for the *argU*, *ileY*, *leuW,* and *proL* tRNAs via constitutive expression in pACYC-RIPL blocked Hha repression of type I fimbriae as demonstrated by increased yeast agglutination (Figure 3).

There are implications for this mechanism for the Hha control of virulence genes since usually they contain a high proportion of rare codons due to their acquisition by horizontal transfer [61]. Hence, Hha-regulated genes may contain an abundance of rare codons so that they may be repressed by Hha at the translational level by the mechanism we described for the fimbrial genes. In support of this, *hilA*, which controls the virulence genes of the pathogenicity island SPI-1 of *S. enterica*, is repressed by Hha [16], and our analysis shows it contains an abundance of rare codons including isoleucine ATA (14-fold more than the expected), arginine AGA (31-fold), proline CCC (7-fold), and leucine TTG (6-fold). Therefore, the Hha-rare tRNA translational regulatory mechanism may be important for the regulation of this gene. Another example is that efficient expression of hemolysin encoded by *hlyA* in uropathogenic *E. coli* is dependent on the availability of *leuX* [62], and we observed that *hlyA* has rare codons for *argU* (5-fold more than expected AGA and 6.6-fold more AGG), for *ileX* and *ileY* (3-fold more ATA), for *proL* (1.53-fold more CCC), and for *thrU* (2.5-fold more ACA). Since Hha is a well-known transcriptional hemolysin repressor, it is also possible that the diminution of the availability of these regulatory tRNAs also plays an important role in Hha-mediated hemolysin repression. In addition it was found recently in *Salmonella* that Hha and its paralog YdgT modulate the transcription mainly of horizontally-acquired genes [63]; hence, Hha may be a key regulator of virulence factors at both the transcriptional and translational level as is shown for type I fimbriae genes in this study.

Our data also suggest that Hha may be part of a toxin-antitoxin pair with YbaJ (Figure 6B); this hypothesis could be confirmed by evaluating Hha-YbaJ protein-protein interactions. One of our main findings is that Hha toxicity stems from cell lysis (Figure S1), and that at least part of the toxic Hha effect is caused by the depletion of regulatory tRNAs encoding rare codons (Figure 6A). Our data also indicate that the mechanism by which Hha is toxic may be due to activation of the DLP12 and CP4-57 prophage lytic genes that are nearby the Hha binding sites *argU* and *ileY* (Table S6), and also by the indirect activation of several toxins by the degradation of their corresponding antitoxins by Lon, ClpP, and ClpX proteases and that the rare codon tRNAs repressed by Hha may trigger the activation of these lytic genes and proteases. Hence, cryptic phage genes may be retained by the cell for lysis and are regulated in part by Hha.

One of the most surprising results was that Hha overexpression triggers the formation of lytic plaques; however, the detailed mechanism is a subject for further research. So far we have many promising links between phage activation and Hha; for example, some of the prophage genes that Hha regulates are related to Hha toxicity and some of those genes, like *alpA* and *ihfA*, have a role in phage activation (*alpA* is associated with the excision of CP4-57 [45], and IHF is essential for the lysogenic life cycle of lambda phage [64]). It is possible that *ssrA* (a tmRNA part of the CP4-57 prophage locus that regulates the expression of many phage) may be under the control of Hha (like the rare codon tRNAs) and both are involved in the activation of phage and cell lysis. Additionally, stress conditions, like starvation, trigger the activation of the lambda phage lytic cycle; hence, the stress produced by Hha overexpression may activate the production of active phage.

YbaJ may exert part of its attenuation of Hha toxicity by repressing these prophage lytic genes since it binds near lytic genes (e.g., *sfmH*) and in lytic genes (e.g., *ybcS*) (Table S3). Note biofilm formation by the *ybaJ* strain in LB glu medium after 24 h was the same as the *hha* mutant (data not shown); hence, deleting *ybaJ* likely inactivates *hha* and leads to the observed increase in biofilm formation. Due its important induction in biofilms and its role in counteracting Hha induced toxicity (that may be mediated by the expression of multiple phage lytic genes and toxins like *essD*, *ybcS*, *rzpD* and *ypjF*), we propose *ybaJ* should be named *tomB* (toxin overexpression modulator in biofilms).

The regulation of cell lysis in biofilms by Hha-YbaJ may be an important aspect of the *E. coli* biofilm physiology since in the biofilms of *Pseudomonas aeruginosa* and *Pseudoalteromonas tunicate,* cell lysis is either mediated by the activation of prophage genes or by autolytic proteins, and these events promote biofilm dispersion and enhance phenotypic variation that may be important for the successful adaptation and colonization to new niches [49]. Therefore, in *E. coli* biofilms, cell death mediated by Hha may also influence these phenotypes. Furthermore, our results suggest another important function of toxin-antitoxin pairs in bacterial physiology in addition to the nine already proposed [42]: these proteins may also induce biofilm dispersal in response to the environmental conditions. The activation of prophage genes for this cell lysis, as controlled by the availability of rare tRNAs, may have additional biological relevance since biofilms are exposed to nutrient (amino acid) starvation which would lead to the diminution of charged rare tRNAs, and this may activate the prophage killing genes allowing the starving cells to commit suicide which would diminish competition for nutrients with nearby cells, provide nutrients to other cells, promote biofilm dispersal (which also is induced by starvation in *Pseudomonas putida* [65]), and promote phenotypic variation. A toxin-antitoxin module controlling this process may be a good strategy for the cells to trigger cellular death in response to starvation, since upon starvation, protein synthesis will diminish (including the synthesis of the toxin-antitoxin proteins) and since the toxin half-life is greater than the antitoxin half-life, after protein synthesis is diminished, the toxin is free to cause cell death. The fact that Hha expression promotes dispersal via toxicity and via repression of fimbriae may be the physiological reason that explains the apparent paradox of induction of Hha, a biofilm inhibitor, in biofilms, since its function may be to promote the dispersal of those biofilms and to allow the detached cells to stay unattached to promote colonization in distant niches.

## Materials and Methods

### Bacterial strains and growth media

The *E. coli* strains and plasmids are listed in Table S5. *E. coli* K-12 BW25113, its single isogenic mutants, *E. coli* AG1, and pCA24N and its derivatives were obtained from the Genome Analysis Project in Japan [66], [67]. *hha, fimA,* and *ihfA* in the pCA24N constructs were tightly-regulated with the *lac* promoter and its repressor *lacI^q^* by the addition of isopropyl-β-*D*-thiogalactopyranoside (IPTG, Sigma, St. Louis, Mo.). The promoter-probe vector plasmid pPROBE-*gfp*[tagless] was provided by Dr. Lindow [37] and was used to construct pP*fimA*-*gfp* containing the *fimA* promoter-*gfp* fusion, pP*ihfA*-*gfp* containing the *ihfA* promoter-*gfp* fusion, and pP*ybaJ*-*gfp* containing the *ybaJ-hha* promoter-*gfp* fusion. The expression vector plasmid pBAD-*Myc*-His C (Invitrogen, Carlsbad, CA) was used to construct pBAD-*hha* and pBAD-*ybaJ*. Expression of Hha or YbaJ proteins under the arabinose-inducible *araBAD* promoter (*P_BAD_*) was induced by 0.1% *L*-arabinose (Acros Organics, Morris Plains, NJ). The broad host range, IPTG-inducible plasmid pVLT31 was provided by Dr. de Lorenzo [68] and was used to construct pVLT31-*hha*. pACYC-RIPL, a plasmid carrying the tRNA genes *argU*, *ileY*, *leuW*, and *proL,* was isolated from BL21-CodonPlus cells (Stratagene, La Jolla, CA).

All experiments were conducted at 37°C. Luria-Bertani medium (LB) [69] was used to pre-culture all the *E. coli* strains with appropriate antibiotics to maintain plasmids. LB and LB supplemented with 0.2% (wt/vol) glucose (LB glu) were used for the crystal-violet biofilm experiments. LB was also used for the growth rate assay, GFP fluorescence intensity assays, and the yeast agglutination assay. LB and LB glu were used for the glass wool biofilm DNA microarrays, and LB glu was used for the nickel-enrichment DNA microarrays, for the indole determination assay, and for the biofilm dispersal experiment with flow cells. Kanamycin (50 µg/mL) was used for pre-culturing all of the *E. coli* BW25113 isogenic mutants, and kanamycin (100 µg/mL), chloramphenicol (30 µg/mL), ampicillin (100 µg/mL), and tetracycline (20 µg/mL) were used to maintain plasmids (for two-plasmid systems, multiple antibiotics were used at the same concentrations). Cells were pre-cultured overnight at 37°C from single colonies with shaking (250 rpm) for the various assays and experiments (biofilm, indole, motility, planktonic growth, Hha toxicity, cell lysis, biofilm DNA microarrays, and nickel-enrichment DNA microarrays). The specific growth rates were measured using cell turbidity at 600 nm using two independent cultures (turbidity less than 0.7).

### *E. coli* double mutants

The *hha/fimA*, *hha/ihfA*, *hha/tqsA*, and *hha/bssS* double mutants were constructed as described previously [70] through bacteriophage P1 transduction to transfer various deletions from the KEIO collection [66]. The kanamycin-resistance gene was deleted using FLP recombinase by electroporating pCP20 (Cm^R^) [70], [71].

### Plasmid construction

To construct the *fimA* promoter-*gfp* fusion, a 585 bp DNA fragment containing the *fimA* promoter region (from -563 bp to -1 bp upstream of the start codon of *fimA*) including the 314 bp switching element and the two inverted repeat sequences IRF and IRR was obtained by PCR amplification using BW25113 genomic DNA as template with primers 5′-CCCCGAGCTCGCTGCTTTC CTTTCAAAAAA-3′ and 5′-CCCCGGATCCCCGCCAGTAATGCTGCTCGT-3′ that form *Bam*HI and *Sac*I restriction sites flanking the *fimA* promoter. Similarly, to construct the *ihfA* promoter-*gfp* fusion, a 314 bp DNA fragment containing the *ihfA* promoter region (from -296 bp to -4 bp upstream of the start codon of *ihfA*) was obtained by PCR with primers 5′-CCCCGGATCCGATTTCTCGCTTCCCGGCGA-3′ and 5′-CCCCGAGCTCGTTCAATCCCTCAATGATGC-3′. Similarly, to construct the *ybaJ*-*hha* promoter-*gfp* fusion, a 220 bp DNA fragment containing the *ybaJ*-*hha* promoter region (from -198 bp to -1 bp upstream of the start codon of *ybaJ*) was obtained by PCR with primers 5′-CCCCGGATCCGCTGC GGGTTAGTGCTAGTA-3′ and 5′-CCCCGAGCTCGAACGCGTCCCCTTCTTAGCG-3′. After double digestion with *Bam*HI and *Sac*I, the DNA fragments were cloned into the promoter-probe vector plasmid pPROBE-*gfp*[tagless] [37] to obtain the pP*fimA*-*gfp*, pP*ihfA*-*gfp,* and pP*ybaJ*-*gfp*.

To construct the pBAD-*hha* plasmid, a 237 bp DNA fragment containing the *hha* gene (from +4 bp to +216 bp downstream of the start codon of *hha*) was obtained by PCR amplification using BW25113 genomic DNA as the template with primers 5′-CCCCCTCGAGCTCCGAAAAACCTTTAAC GAA-3′ and 5′-CCCCTTCGAATTAGCGAATAAATTTCCATAC-3′ that form *Xho*I and *Hind*III restriction sites flanking the *hha* gene. Similarly, to construct pBAD-*ybaJ*, a 393 bp DNA fragment containing the *ybaJ* gene (from +4 to +372 bp downstream of the start codon of *ybaJ*) was obtained by PCR amplification using BW25113 genomic DNA as template with primers 5′-CCCCCTCGAGCGATGAATACTCACCC AAAAG-3′ and 5′-CCCCTTCGAACTAACAAGATAAACTCGCAG-3′ that form *Xho*I and *Hind*III restriction sites flanking the *ybaJ* gene. After double digestion with *Xho*I and *Hind*III, the DNA fragments were cloned into pBAD-*Myc*-His C to obtain pBAD-*hha* and pBAD-*ybaJ*, which expresses Hha and YbaJ, respectively, under the *L*-arabinose-inducible P_BAD_ promoter.

To construct pVLT31-*hha* plasmid, a 248 bp DNA fragment containing the *hha* gene (from -5 bp upstream to +222 bp downstream of the start codon of *hha*) was obtained by PCR amplification using BW25113 genomic DNA as the template with primers 5′-GCGAATTCGAAGTATGTCCG-3′ and 5′-GCGCTCTAGATATTTATTAGCG-3′ that form *EcoRI* and *Xba*I restriction sites flanking the *hha* gene.

### Crystal-violet biofilm and indole assays

The biofilm assay was conducted in 96-well polystyrene plates [35]. Bacteria were inoculated at an initial turbidity at 600 nm of 0.05 in all media at 37°C or 30°C for 4 h, 7 h, 15 h, and 24 h without shaking, then the growth (turbidity at 620 nm) and total biofilm were measured using crystal violet staining. IPTG addition (2 mM) had no effect on biofilm formation of the wild type strain in both LB and LB glu media. Each data point was averaged from at least twenty replicate wells using at least four independent cultures of each strain. Extracellular indole concentrations of BW25113 and the *hha* mutant cultured in LB glu were measured spectrophotometrically as described [25] at 15 h. Two independent cultures were used for each strain.

### Yeast agglutination assay

The evaluation of type I fimbriae production was measured via yeast agglutination as described [34]. Briefly, bacteria were grown for 24 h at 37°C on LB plates with appropriate antibiotics to select plasmids. For the induction of complemented genes (*hha*, *fimA*, or *ihfA*) from pCA24N, 0.25 mM IPTG was added, and for the induction of *hha* from pBAD-*hha*, 1% L-arabinose was added. After growth, cells were collected and suspended in phosphate-buffered saline (PBS, NaCl 8 g/L, KCl 0.2 g/L, Na_2_HPO_4_ 1.44 g/L, KH_2_PO_4_ 0.24 g/L, pH 7.4) [69] to a turbidity of 5 at 620 nm and mixed with a 10% w/v brewers yeast suspension (Acros Organics, Morris Plains, NJ). A few drops of the mixture were poured onto microscope glass slides and the yeast agglutination was evaluated after 5 min. Each experiment was performed two times using two independent cultures.

### Hha toxicity

The effect of Hha on cell growth was assayed by inducing Hha expression with 0 to 2 mM IPTG using pCA24N-*hha*. The effect of adding extra copies of the rare codon tRNAs *ileY*, *argU*, *leuW* and *proL* on toxicity was assayed using BL21 (DE3) codon plus-RIPL/pET28A-*hha* which contains rare codon tRNA encoded on pACYC-RIPL; 2 mM of IPTG was added to cells in LB+kanamycin (50 µg/mL)+chloramphenicol (30 µg/mL) to induce Hha expression from pET28A-*hha*. The effect of YbaJ on Hha-mediated toxicity was assayed in BW25113 *hha* by the simultaneous expression of Hha and YbaJ with 2 mM IPTG using pVLT31-*hha* to induce Hha and 0.1% arabinose with pBAD-*ybaJ* to induce YbaJ. The effect of Hha on the cell growth of the prophage mutants was assayed by inducing Hha expression from pCA24N-*hha* with 1 mM IPTG in LB at 37°C.

### Cell lysis plaques

Samples (1 mL) of overnight cultures (cultured with or without 0.5 mM IPTG) were mixed into a soft agar overlay containing 3 mL of media containing 10 g/L tryptone, 1 g/L yeast extract, 8 g/L NaCl, 8 g/L agar, the appropriate antibiotics, and 1 mM IPTG. The overlay was poured onto plates with the same composition but with 10 g/L agar, and the plates were incubated at 37°C for 24 h.

### GFP fluorescence intensity assay

To determine the specific intensity of the GFP fluorescence from the pP*fimA*-*gfp*, pP*ihfA*-*gfp,* and pP*ybaJ*-*gfp* plasmids, the bacterial cultures were grown at 37°C with 250 rpm shaking until late exponential growth phase (turbidity at 600 nm of 1) except for those cultures that contained pCA24N or pCA24N-*hha* that were grown to turbidity of 0.5 at 600 nm and then 0.5 mM IPTG was added and the incubation was continued for two hours. Aliquots were diluted rapidly with PBS to turbidity at 600 nm of 0.1 to 0.2, and the fluorescence was quantified (Spectra Max Germini EM, Molecular Device, Sunnyvale, CA, USA) using excitation at 485 nm and emission at 528 nm with a 515 nm cutoff. For normalization, the final cell density was determined by measuring the turbidity at 600 nm (Tecan, Austria Gesellschaft, Salzburg, Austria). The GFP negative strain BW25113 pPROBE-*gfp*[tagless] was used as the negative fluorescence control. All experiments were performed at least twice.

### Flow cell biofilm experiments and image analysis

LB glu supplemented with erythromycin (300 µg/mL) to maintain the constitutive green fluorescent protein plasmid pCM18 [72] and chloramphenicol (30 µg/mL) to maintain pCA24N-*hha* were used to form biofilms at 37°C in a continuous-flow cell as described previously [27]. The biofilm was visualized at 42 h and at 48 h (after 6 h of the IPTG addition) with a TCS SP2 scanning confocal laser microscope (Leica Microsystems, Heidelberg, Germany) with a 40 X N PLAN L dry objective with a correction collar and a numerical aperture of 0.55. Confocal flow cell images were analyzed with COMSTAT image-processing software [73] as described previously [74].

### RNA isolation and DNA microarrays

Biofilm cells on glass wool for each strain for the four sets of DNA microarrays were prepared as described previously [20]. Suspension cells of BW25113/pCA24N and BW25113/pCA24N-*hha* were cultured in 25 mL of LB at 37°C supplemented with 2 mM IPTG (initial turbidity 0.1 at 600 nm) to a turbidity at 600 nm of 0.5. Total RNA was isolated from cells as described [18]. The *E. coli* Genechip antisense genome array chip (P/N 900381, Affymetrix, Santa Clara, CA) was used to analyze the complete *E. coli* transcriptome of biofilm cells, and the *E. coli* Genechip Genome 2.0 array chip (P/N 900550, Affymetrix, Santa Clara, CA) was used to analyze the suspension cells as described [20]; note that there were no comparisons of data from different Genechips. Background values, noise values, and scaling factors of all arrays were examined and were comparable. The intensities of polyadenosine RNA controls were used to monitor the labeling process. As expected, signals of the deleted genes, *araA* and *rhaA,* were low for all strains, while the signal of *hha* was low for strains with *hha* deleted and high for strains were *hha* was overexpressed. For both sets of binary microarray comparisons to determine differential genes expression, if the gene with the larger transcription rate did not have a consistent transcription rate based on the 11-15 probe pairs (*p-*value less than 0.05), these genes were discarded. A gene was considered differentially expressed when the *p*-value and the corrected *p*-value based on the False Discovery Rate Method [75] for comparing two chips were lower than 0.05 (to assure that the change in gene expression was statistically significant and that false positives arise less than 5%) and when the expression ratio was higher than the standard deviation for all of the *E. coli* K-12 genes (1.4 for the 4 h LB, 1.4 for the 4 h LB glu, 3.3 for the 15 h LB glu, 1.6 for the 24 h LB glu, and 1.8 for the suspension cell arrays) [76].

### Microarray accession numbers

The expression data for biofilm samples of the *E. coli* wild type and *hha* mutant, and the expression data of suspension cells upon Hha overexpression have been deposited in the NCBI Gene Expression Omnibus (GEO) [77] and are accessible as GSE8708 (for biofilm cells) and GSE10116 (for Hha overexpression with suspension cells).

### Quantitative Real Time PCR

RT-PCR was performed using an iCycler (Bio-Rad, CA). Approximately 50 ng of total RNA from the wild type strain, *hha* mutant, or *hha* mutant harboring pCA24N-*hha* with and without the addition of 0.5 mM IPTG were used for reverse transcription. Planktonic, exponentially-growing cells with a turbidity at 600 nm of 1, cultured in LB at 37°C with shaking (250 rpm) or glass wool biofilm cells cultured for 15 h in LB glu at 37°C with shaking (250 rpm) were used. Primers used to amplify *argU* (77 bp), *proL* (76 bp), *ileV* (77 bp), *leuP* (87 bp), *argQ* (77 bp), a fragment of *fimA* (202 bp), a fragment of *ihfA* (164 bp), a fragment of *ybaJ* (204 bp), a fragment of *lon* (219 bp), a fragment of *dinJ* ( 219 bp), a fragment of *yfeM* (208 bp), and a fragment of the housekeeping *rrsG* gene (234 bp) were 5′-GCGCCCTTAGCTCAGTTGGA-3′/5′-TGGCGCGCCCTGCAGGATTC-3′, 5′-CGGCACGTAGCG CAGCCTGG-3′/5′-TGGTCGGCACGAGAGGATTT-3′, 5′-AGGCTTGTAGCTCAGGTGGT-3′/5′-TGGTAGCCTGAGTGGACTT-3′, 5′-GCGAAGGTGGCGGAATTGGT-3′/5′-TGGTG CGAGGGGGGGGACTT-3′, 5′-GCATCCGTAGCTCAGCTGGA-3′/5′-TGGTGCATCCGGGAGGAT TC-3′, 5′-CTCTGGCAATCGTTGTTCTG-3′/5′-CTGGTTGCTCCTTCCTGTGC-3′, 5′-ATGGCGCTT ACAAAAGCTGA-3′/5′-CGCAGATCGAAGTTACCAAA-3′, 5′-GATGAATACTCACCCAAAAG-3′/5′-TCTTCATTATACTTAATTT-3′, 5′-CGTGCGTGATGAAGCGGAA-3′/5′- AGGTAGTGGTCGCTGAACG-3′, 5′-GCTGCTAACGCGTTTGTTC-3′/5′- GGCCTTATGAACATCAATGC-3′, 5′-CGTACAATTAGCTACAGCGA-3′/5′-GGCTATCGATTGAGTCCATC-3′, and 5′-TATTGCACAATGGGCGCAAG-3′/5′-ACTTAACAA ACCGCTGACTT-3′, respectively. Threshold cycle numbers were calculated using the MyiQ software (Bio-Rad), and PCR products were verified using agarose electrophoresis.

### *fimA* switch orientation assay

PCR was conducted using genomic DNA extracted from exponentially-grown wild-type and *hha* mutant cells (cell turbidity at 600 of 1). The primers used were 5′-GCCGGATTATGGGAAAGA-3′ and 5′-AGCCGCTGTAGAACTGAGGG-3′ [38]. The amplified region (604 bp) was digested with *AluI*, which produces fragments of 258 bp+346 bp for the promoter in the on orientation and of 510 bp+94 bp for the promoter in the off orientation. The restriction digested fragments were separated on polyacrylamide gradient (4-20%) gels.

### Nickel-enrichment DNA microarrays

These experiments were performed as reported previously [39]. Briefly, overnight cultures of *E. coli* K-12 BW25113 *hha*/pCA24N (control) and BW25113 *hha*/pCA24N-*hha*, BW25113 *hha hns*/pCA24N-*hha,* or AG1/pCA24N-*ybaJ* (expressing Hha or YbaJ with a His_6_-tag at the amino terminus) [67] were diluted into 250 mL of LB glu media with chloramphenicol (30 µg/mL) to maintain pCA24N, pCA24N-*hha*, and pCA24N-*ybaJ* and with 1 mM IPTG to induce Hha or YbaJ synthesis. Cultures were grown at 37°C with 250 rpm shaking in 1 L capacity Erlenmeyer flasks containing 10 g glass wool to produce biofilms [18]. After 24 h, formaldehyde (1%, Fisher Scientific Co., Pittsburgh, PA) was added to crosslink the His-tagged Hha or YbaJ proteins and the DNA to which it was bound (present also were proteins like H-NS that may interact with Hha *in vivo*). Ni-NTA agarose gel resin was used to bind the His_6_-tagged Hha-DNA or His_6_-tagged YbaJ-DNA complexes from the biofilms cells, and RNase A and proteinase K were used to remove RNA and protein. The DNA fragments were labeled [18], and the DNA microarrays were performed as indicated above. Positive candidates for the Hha or YbaJ binding *in vivo* were those genes or intergenic regions with at least a 4-fold higher signal than the global average signal of all the genes and IG regions in the Hha or YbaJ chips (∼600), and those that were at least 15-fold enriched with respect to the signal in the control array (the pCA24N vector). This cut off ratio was selected because the standard deviation of the enrichment (Hha or YbaJ signal divided by empty vector signal) was 1.3 for the Hha experiment and 5.3 for the YbaJ experiment. The spiked control *rhaD* gene gave a signal from ∼13,000 to ∼32,000 in the experiments (∼22-fold to ∼54-fold higher than the average signal) which validated our methodology.

## Supporting Information

Figure S1 Inhibition of growth in LB at 37°C by Hha overexpression via pCA24N-*hha* is less severe in various mutants: (A) *rzpD*, (B) *yfjZ*, (C) *alpA*, (D) *appY*, and (E) *clpP/clpX*. Each experiment was performed three times, and one representative experiment is shown.
(1.81 MB TIF)

Figure S2 Plaques in soft agar containing 1 mM IPTG upon expression of Hha at 37°C in BW25113 *hha*/pCA24N-*hha*. BW25113 *hha*/pCA24N was used as the negative control and the effect of 0.5 mM IPTG to induce Hha in the overnight culture is shown. The experiment was performed in duplicate, and one representative experiment is shown.
(2.45 MB TIF)

Table S1 *E. coli* BW25113 genes *induced* more than two fold (P<0.05) in biofilms upon deleting *hha* in at least two of the following conditions: LB 4 h, LB glu 4 h, LB glu 15 h, and LB glu 24 h.
(0.12 MB DOC)

Table S2 *E. coli* BW25113 genes *repressed* more than two fold (P<0.05) in biofilms upon deleting *hha* in at least two of the following conditions: LB 4 h, LB glu 4 h, LB glu 15 h, and LB glu 24 h.
(0.08 MB DOC)

Table S3 List of gene regions bound *in vivo* by Hha and YbaJ in biofilms cells at 37°C in LB glu after 24 h as identified by nickel-enrichment DNA microarrays in *E. coli* K12 BW25113 *hha*, *E. coli* K12 BW25113 *hha/hns*, and *E. coli* K12 AG1. Signal ratio indicates fold change of pCA24N-*hha* or pCA24N-*ybaJ* cells relative to cells with the empty plasmid pCA24N. ^1^Gene or intergenic region close to *argU*. ^2^Gene or intergenic region close to *ileY*. ^3^Gene or intergenic region close to *appY*. ^4^Prophage gene or intergenic regions next to prophage genes.
(0.12 MB DOC)

Table S4 Rare codons present in *E. coli fim* genes. The numbers in bold indicate more rare codons than expected according the *E. coli* codon usage for the complete genome. The expected use of rare codons in E. coli is as follows: 7% of all the isoleucine codons should be ATA, 13% of all the proline codons should be CCC, 4% of all the arginine codons should be AGA, 2% of all the arginine codons should be AGG, 13% of all the leucine codons should TTG, 4% of all the leucine codons should be CTA, and 13% of all the threonine codons should be ACA. The codon usage data were obtained from the Genomic Atlas Database (http://www.cbs.dtu.dk/services/GenomeAtlas/).
(0.07 MB DOC)

Table S5 *E. coli* strains and plasmids used. Km^R^, Cm^R^, Em^R^, Sm^R^ and Amp^R^ are kanamycin, chloramphenicol, erythromycin, streptomycin, and ampicillin resistance, respectively.
(0.11 MB DOC)

Table S6 *E. coli* DLP12 and CP4-57 prophage genes that are differentially regulated (P<0.05) in BW25113 biofilms upon deleting *hha* in LB glu after 15 h and in LB glu after 24 h.
(0.04 MB DOC)

Table S7 *E. coli* genes *induced* and *repressed* more than 4 fold (P<0.05) in exponentially-growing suspension cells (turbidity at 600 nm of 1) upon *hha* overexpression in *E. coli* K12 BW25113/pCA24N-*hha* in LB medium with 2 mM IPTG at 37°C.
(0.11 MB DOC)
