# Supplementary figures and images for: Protein Translation and Cell Death: The Role of Rare tRNAs in Biofilm Formation and in Activating Dormant Phage Killer Genes
Source: PLoS One. 2008 Jun 11;3(6):e2394. doi: 10.1371/journal.pone.0002394 (PMC2408971; doi:10.1371/journal.pone.0002394)

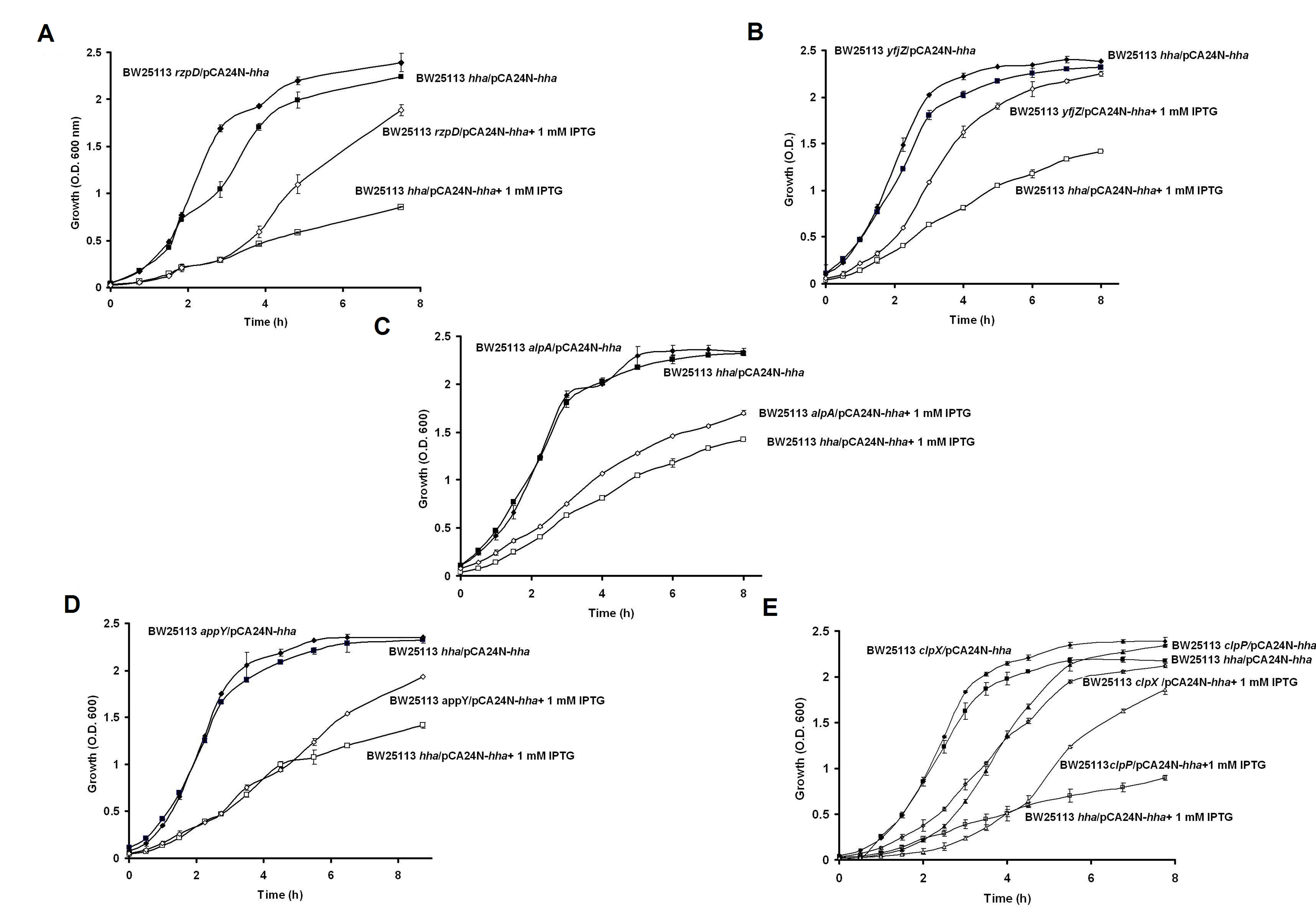

Supplement: Figure S1 — Inhibition of growth in LB at 37°C by Hha overexpression via pCA24N-hha is less severe in various mutants: (A) rzpD, (B) yfjZ, (C) alpA, (D) appY, and (E) clpP/clpX. Each experiment was performed three times, and one representative experiment is shown. (1.81 MB TIF) [file pone.0002394.s001.tif]

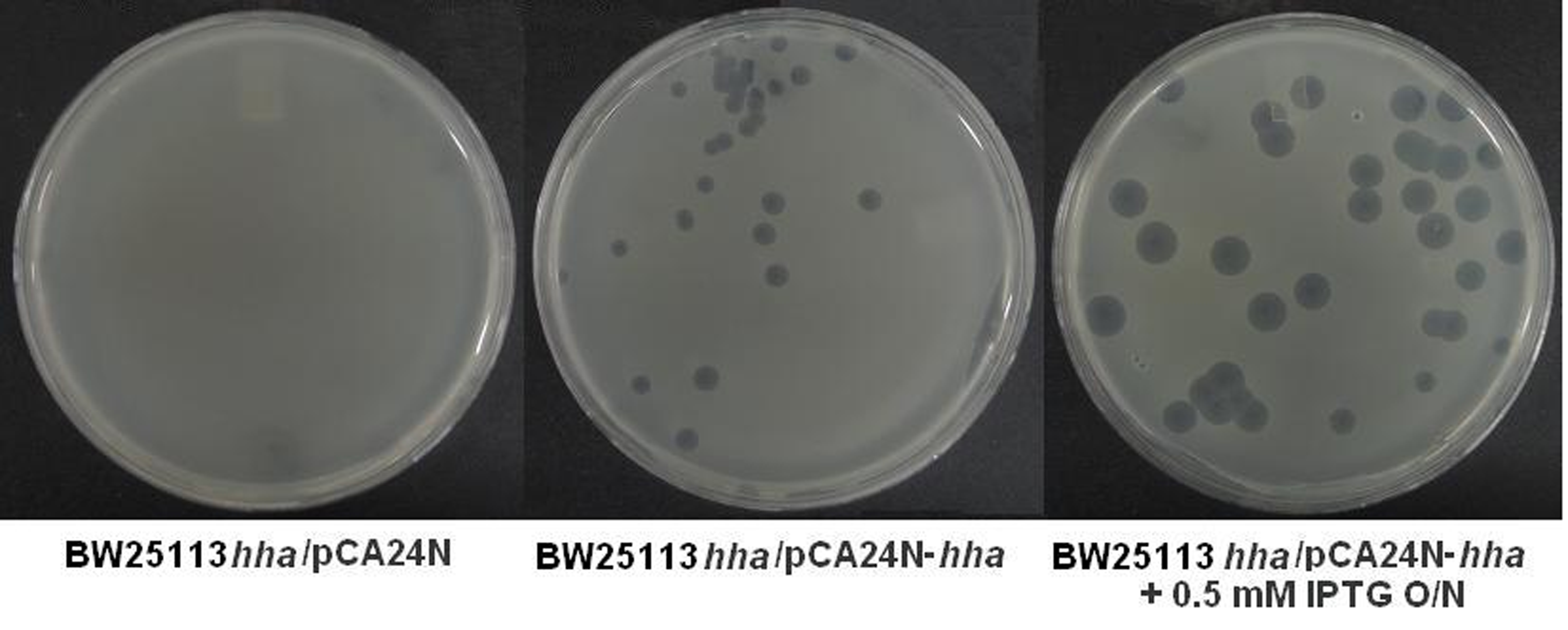

Supplement: Figure S2 — Plaques in soft agar containing 1 mM IPTG upon expression of Hha at 37°C in BW25113 hha/pCA24N-hha. BW25113 hha/pCA24N was used as the negative control and the effect of 0.5 mM IPTG to induce Hha in the overnight culture is shown. The experiment was performed in duplicate, and one representative experiment is shown. (2.45 MB TIF) [file pone.0002394.s002.tif]
