# Supplementary material for: Protein Translation and Cell Death: The Role of Rare tRNAs in Biofilm Formation and in Activating Dormant Phage Killer Genes
Source: PLoS One. 2008 Jun 11;3(6):e2394. doi: 10.1371/journal.pone.0002394 (PMC2408971; doi:10.1371/journal.pone.0002394)
Supplement: Table S1 — E. coli BW25113 genes induced more than two fold (P<0.05) in biofilms upon deleting hha in at least two of the following conditions: LB 4 h, LB glu 4 h, LB glu 15 h, and LB glu 24 h. (0.12 MB DOC) [file pone.0002394.s003.doc]

**Supporting Table S1.** *E. coli* BW25113 genes induced more than two fold (P < 0.05) in biofilms upon deleting *hha* in at least two of the following conditions: LB 4 h, LB glu 4 h, LB glu 15 h, and LB glu 24 h.

| **Gene** | **B number** | **Induction**  **4 h LB** | **Induction 4 h LB glu** | **Induction 15 h LB glu** | **Induction 24 h LB glu** | **Description** |
| --- | --- | --- | --- | --- | --- | --- |
| **RNA** |  |  |  |  |  |  |
| *rrfA* | b3855 |  |  | 4 | 2.6 | 5S ribosomal RNA |
| *rrlc* | b3758 |  |  | 4.3 | 2.4 | 23S ribosomal RNA |
| **Regulator** |  |  |  |  |  |  |
| *csrA* | b2696 |  |  | 5 | 2.4 | Carbon storage regulator |
| *ihfA* (*himA*) | b1712 |  | 2 | 4.6 | 4 | Subunit of IHF transcriptional dual regulator |
| *yiaG* | b3555 |  | 2 | 6.5 |  | Predicted transcriptional regulator |
| *bssR* | b0836 | 2.4 |  | 4.6 |  | Regulator of biofilm formation |
| **Stress response** |  |  |  |  |  |  |
| *csiD* | b2659 |  | 2.8 |  | 3 | Predicted protein, induced by carbon starvation |
| *csiE* | b2535 |  |  | 4.6 | 2.4 | Stationary phase inducible protein |
| *dps* | b0812 |  | 2 | 4 | 2.8 | Stationary phase nucleoid component that sequesters iron and protects DNA from damage |
| *gadC* | b1492 | 4.9 |  | 5.1 |  | Glutamic acid:γ-aminobutyrate antiporter |
| *hdeD* | b3511 | 2.4 |  | 4.3 | 2.4 | Acid-resistance membrane protein |
| *hspQ* | b0966 |  | 2.3 | 6.5 |  | Heat shock protein |
| *osmC* | b1492 |  |  | 4 | 3.5 | Osmotically inducible peroxidase |
| *osmE* | b1739 |  | 2 | 5 | 4.3 | Osmotically inducible protein |
| *osmY* | b4376 |  | 3 | 5.6 |  | Hyperosmotically inducible periplasmic protein |
| *slp* | b3506 | 2.6 |  | 4 |  | Starvation lipoprotein |
| *hchA* | b1967 |  | 3 |  | 2.5 | Hsp31 molecular chaperone |
| *ybaY* | b0453 |  | 2.5 | 7.4 | 3 | Predicted outer membrane lipoprotein associated with the osmotic stress response |
| *ygaM* | b2672 |  |  | 5.2 | 2.5 | Putative osmoprotectant protein |
| **Flagella and Fimbriae** |  |  |  |  |  |  |
| *fimA* | b3414 | 2.3 | 2 |  |  | Major type I fimbriae subunit |
| *fliC* | b1923 | 2.1 | 2.8 |  |  | Flagellin, basic subunit of flagellar filament |
| **Metabolism** |  |  |  |  |  |  |
| *acnA* | b1276 |  | 2.8 |  | 2.8 | Aconitase (glyoxylate cycle) |
| *fbaB* | b2097 |  | 2.1 |  | 2.6 | Fructose bisphosphate aldolase |
| *prpB* | b0331 |  |  | 36 | 5.3 | 2-Methylisocitrate lyase |
| *prpC* | b0333 |  |  | 9 | 2.5 | Subunit of methylcitrate synthase |
| *prpD* | b0334 |  |  | 11 | 3.7 | 2-Methylcitrate dehydratase |
| *sucA* | b0726 |  | 5.6 |  | 3 | 2-Oxoglutarate decarboxylase |
| *talA* | b2464 |  | 2.1 |  | 2.6 | Transaldolase A |
| *thrL* | b0001 |  | 3.2 |  | 7.4 | Thr operon leader peptide |
| *wrbA* | b1004 |  | 2 | 4 | 2.4 | NAD(P)H:quinone oxidoreductase |
| *rnpB* | b3123 |  |  | 4 | 3 | Catalytic subunit of RNAse P |
| *tktB* | b2465 |  | 2.1 |  | 2.4 | Transketolase |
| **Unknown function** |  |  |  |  |  |  |
| *elaB* | b 2266 |  | 2.1 | 4 |  | Hypothetical protein |
| *yahO* | b0329 |  | 2.1 | 5.6 |  | Predicted protein |
| *ybaJ* | b0461 | 2.1 | 3.2 | 9.8 | 6.5 | Predicted protein |
| *ynhG* | b1678 |  | 2 | 8 | 2.4 | Conserved protein |
| *yodD* | b1953 |  | 3.2 | 4.6 | 2.8 | Predicted protein |
| *ygdI* | b2809 |  | 3 | 4 |  | Putative lipoprotein |
| *yhiM* | b3491 | 2.6 | 2.1 |  |  | Conserved inner membrane protein |
| *ybgS* | b0753 |  | 2.5 | 7.4 | 3 | Putative homeobox protein |
| *ycfH* | b1100 |  | 2.3 | 9.1 |  | Predicted metallodependent hydrolase |
| *ycgB* | b1188 |  | 2.1 | 4.3 |  | Putative sporulation protein |
| *yeaG* | b1783 |  | 2 | 4.9 |  | Conserved protein |
