# Supplementary material for: Protein Translation and Cell Death: The Role of Rare tRNAs in Biofilm Formation and in Activating Dormant Phage Killer Genes
Source: PLoS One. 2008 Jun 11;3(6):e2394. doi: 10.1371/journal.pone.0002394 (PMC2408971; doi:10.1371/journal.pone.0002394)
Supplement: Table S2 — E. coli BW25113 genes repressed more than two fold (P<0.05) in biofilms upon deleting hha in at least two of the following conditions: LB 4 h, LB glu 4 h, LB glu 15 h, and LB glu 24 h. (0.08 MB DOC) [file pone.0002394.s004.doc]

**Supporting Table S2.** *E. coli* BW25113 genes repressed more than two fold (P < 0.05) in biofilms upon deleting *hha* in at least two of the following conditions: LB 4 h, LB glu 4 h, LB glu 15 h, and LB glu 24 h.

| **Gene** | **B number** | **Repression**  **4 h LB** | **Repression 4 h LB glu** | **Repression 15 h LB glu** | **Repression 24 h LB glu** | **Description** |
| --- | --- | --- | --- | --- | --- | --- |
| **RNA** |  |  |  |  |  |  |
| *proM* | b3799 |  | -2.2 | -11 |  | Proline tRNA |
| **Fimbriae** |  |  |  |  |  |  |
| *fimD* | b4317 |  |  | -4 | -3 | Usher constituent in the type I fimbria chaperone |
| **Metabolism** |  |  |  |  |  |  |
| *agaW* | b3134 |  |  | -6 | -5.6 | Enzyme IIB^Aga^ of the N-acetyl-galactosamine phosphotransferase system |
| *ansB* | b2957 |  | -2.5 | -6 |  | Asparaginase II |
| *cysC* | b2750 |  | -3.2 | -4 |  | Adenylylsulfate kinase |
| *nirC* | b3367 |  |  | -8.5 | -8 | Nitrite FNT transporter |
| *sieB* | b1353 |  |  | -4 | -2.6 | Phage superinfection exclusion protein |
| *srlE* | b2703 | -2 |  | -8.5 | -3.7 | Glucitol/sorbitol-specific enzyme IIB component of PTS |
| *tdcB* | b3117 |  | -3.2 | -5 |  | Threonine dehydratase |
| *tdcD* | b3115 |  | -2.2 |  | -2.4 | Propionate kinase / acetate kinase C |
| *yeeU* | b2004 | -2 |  |  | -2.6 | Antitoxin of the YeeV-YeeU toxin-antitoxin system |
| **Unknown function** |  |  |  |  |  |  |
| *ydaC* | b1347 |  |  | -5 | -5 | Hypothetical protein |
| *ygeL* | b2856 |  |  | -4.6 | -2.4 | Predicted protein |
| *yieL* | b3719 |  | -2.1 | -8 |  | Putative xylanase |
| *ykgE* | b0306 |  | -2.5 | -7 |  | Predicted oxidoreductase |
| *ylbE* | b4507 |  |  | -9.1 | -2.6 | Predicted protein |
| *yihN* | b3874 |  |  | -9.8 | -5.6 | Predicted proton-driven sugar phosphate uptake system |
| *yjhQ* | b4307 |  |  | -5 | -2.4 | Predicted acetyltransferase |
