# Supplementary material for: Protein Translation and Cell Death: The Role of Rare tRNAs in Biofilm Formation and in Activating Dormant Phage Killer Genes
Source: PLoS One. 2008 Jun 11;3(6):e2394. doi: 10.1371/journal.pone.0002394 (PMC2408971; doi:10.1371/journal.pone.0002394)
Supplement: Table S3 — List of gene regions bound in vivo by Hha and YbaJ in biofilms cells at 37°C in LB glu after 24 h as identified by nickel-enrichment DNA microarrays in E. coli K12 BW25113 hha, E. coli K12 BW25113 hha/hns, and E. coli K12 AG1. Signal ratio indicates fold change of pCA24N-hha or pCA24N-ybaJ cells relative to cells with the empty plasmid pCA24N. 1Gene or intergenic region close to argU. 2Gene or intergenic region close to ileY. 3Gene or intergenic region close to appY. 4Prophage gene or intergenic regions next to prophage genes. (0.12 MB DOC) [file pone.0002394.s005.doc]

**Supporting Table S3.** List of gene regions bound *in vivo* by Hha and YbaJ in biofilms cells at 37^o^C in LB glu after 24 h as identified by nickel-enrichment DNA microarrays in *E. coli* K12 BW25113 *hha,* *E. coli* K12 BW25113 *hha*/*hns*, and *E. coli* K12 AG1. Signal ratio indicates fold change of pCA24N-*hha* or pCA24N-*ybaJ* cells relative to cells with the empty plasmid pCA24N.

| **Binding gene or intergenic region** | **Signal ratio**  **in *hha* mutant/ pCA24N-*hha*** | **Signal ratio**  **in *hha* *hns* mutant/ pCA24N-*hha*** | **Signal ratio**  **in AG1/** **pCA24N-*ybaJ*** | **Binding gene or intergenic region** | **Signal ratio**  **in *hha* mutant/ pCA24N-*hha*** | **Signal ratio**  **in *hha* *hns* mutant/ pCA24N-*hha*** | **Signal ratio**  **in AG1/** **pCA24N-*ybaJ*** |
| --- | --- | --- | --- | --- | --- | --- | --- |
| *hha* | 5792.6 | 5792.6 |  | *ygeM* | 4.0 |  |  |
| *ybaJ* |  |  | 380.0 | *yhaC-rnpB* | 6.0 |  |  |
| *sfmD-sfmH* ^1^ | 18.4 | 9.8 |  | *yhiK* | 4.0 | 4.6 |  |
| *sfmH* ^1^ |  |  | 13.0 | *yhiL* | 4.0 |  |  |
| *ybcC* ^1, 4^ | 12.1 |  |  | *yhiS-insH-11* | 4.3 | 4.0 |  |
| *ypjB-ypjC* ^2, 4^ | 4.6 | 4.6 |  | *yhiD-hdeB* | 4.0 |  |  |
| *ypjC* ^2, 4^ | 4.0 |  |  | *hdeD-gadE* | 5.2 |  |  |
| *ygaR* ^2^ | 4.0 |  |  | *gadE* |  | 5.6 |  |
| *ygaR-yqaC* ^2^ |  | 4.0 |  | *yqeH-yqeI* | 5.6 | 5.0 |  |
| *ompT-envY* ^3, 4^ | 6.0 | 4.6 |  | *yqeJ-yqeK* | 4.0 |  |  |
| *yagK-yagL*^4^ | 4.0 | 4.3 |  | *ygeF-ygeG* | 5.2 |  |  |
| *intF-yagP* ^4^ | 4.0 | 6.0 |  | *ygeG* | 4.9 |  |  |
| *ydfO* ^4^ | 4.6 | 4.6 |  | *ygeH* | 4.0 |  |  |
| *yfjW* ^4^ | 4.0 | 4.0 |  | *ygeH-ygeI* | 6.5 | 4.0 |  |
| *rzpD* ^4^ | 4.3 |  |  | *rfaJ* |  | 5.0 |  |
| *borD* ^4^ | 8.0 |  |  | *rfaL* | 4.0 |  |  |
| *borD-ybcV* ^4^ | 7.4 |  |  | *rfaY* | 4.6 |  |  |
| *ybcV-ybcW* ^4^ | 9.2 |  |  | *rfaS* | 5.6 | 5.2 |  |
| *ybcW* ^4^ | 9.2 |  |  | *rfaS-rfaP* |  | 5.0 |  |
| *ybcW-ylcI* ^4^ | 6.5 |  |  | *waaU-rfaZ* |  | 5.6 |  |
| *ylcI-nohB* ^4^ | 6.0 |  |  | *ybeF-lipB* | 7.0 | 5.2 |  |
| *ymfE* ^4^ | 4.3 |  |  | *ryhB-yhhY* | 4.0 |  |  |
| *yeeP-isrC* ^4^ | 4.0 |  |  | *yjbM* | 6.0 | 5.0 |  |
| *ybcY-tfaX* ^4^ |  | 4.0 |  | *yjiR-yjiS* | 4.3 |  |  |
| *ybcS* ^4^ |  |  | 18.0 | *yccE* | 4.3 |  |  |
| *yagA* ^4^ |  |  | 12.0 | *elaD* | 4.3 |  |  |
| *yeeO-asnU* | 147.0 | 24.2 |  | *ybjK* |  | 54.0 |  |
| *elbA-ycgX* | 11.3 | 9.2 |  | *ybjG* |  | 10.0 |  |
| *yncI* | 4.6 | 4.6 |  | *ypeC* |  | 9.8 |  |
| *yiaY-selB* | 6.5 | 6.5 |  | *ariR* |  | 4.6 | 9.0 |
| *yibA-yibJ* | 6.5 | 6.5 |  | *ygeI* |  | 4.6 |  |
| *hemB-ykiB* | 4.0 | 5.0 |  | *mqsR-ygiV* |  | 4.0 |  |
| *ybcV* | 14.9 | 5.2 |  | *erfK-cobT* |  | 4.3 |  |
| *ykiB-yaiT* | 4.0 |  |  | *ybhP-ybhQ* |  | 4.0 |  |
| *yaiT-insEF2* |  | 5.0 |  | *mcrA* |  | 4.0 |  |
| *trpL* | 8.0 | 4.6 |  | *rhsB* |  | 4.0 |  |
| *caiF* | 7.4 |  |  | *yddJ* |  | 4.3 |  |
| *lacY* | 6.0 |  |  | *yddK* |  | 4.3 |  |
| *ycgE-ycgF* | 4.0 |  |  | *proM* |  |  | 41.0 |
| *ycgV-ychF* | 4.9 | 6.5 |  | *tufB* |  |  | 40.0 |
| *mokC* | 5.2 |  |  | *atl* |  |  | 32.0 |
| *nhaR* | 4.0 |  |  | *fliD* |  |  | 29.0 |
| *rhsD-ybbC* | 6.0 |  |  | *ymgG* |  |  | 30.0 |
| *ybbD-ylbG* | 4.3 |  |  | *oppA* |  |  | 15.0 |
| *yncI-yncM* | 4.3 |  |  | *oppD* |  |  | 25.0 |
| *cyoA-ampG* | 5.6 | 5.0 |  | *ydeU-ydeK* |  |  | 20.0 |
| *betT-yahA* | 4.0 |  |  | *dinJ* |  |  | 19.0 |
| *gadB-pqqL* | 4.0 |  |  | *mmuP* |  |  | 18.0 |
| *ynfH-dmsD* | 7.0 |  |  | *yfeZ* |  |  | 17.0 |
| *yedN_2* | 4.0 |  |  | *sbcC* |  |  | 12.0 |
| *yaaA* | 5.2 |  |  | *ydbA* |  |  | 11.0 |
| *ydcD* | 4.9 | 4.0 |  | *yiiR* |  |  | 11.0 |
| *ybgD-gltA* | 4.3 | 6.0 |  | *yicO-ade* |  |  | 9.0 |
| *cspE* | 4.9 |  |  | *yaaH* |  |  | 9.0 |

^1^Gene or intergenic region close to *argU*. ^2^Gene or intergenic region close to *ileY*. ^3^Gene or intergenic region close to *appY*. ^4^Prophage gene or intergenic regions next to prophage genes.
