# Supplementary material for: Protein Translation and Cell Death: The Role of Rare tRNAs in Biofilm Formation and in Activating Dormant Phage Killer Genes
Source: PLoS One. 2008 Jun 11;3(6):e2394. doi: 10.1371/journal.pone.0002394 (PMC2408971; doi:10.1371/journal.pone.0002394)
Supplement: Table S4 — Rare codons present in E. coli fim genes. The numbers in bold indicate more rare codons than expected according the E. coli codon usage for the complete genome. The expected use of rare codons in E. coli is as follows: 7% of all the isoleucine codons should be ATA, 13% of all the proline codons should be CCC, 4% of all the arginine codons should be AGA, 2% of all the arginine codons should be AGG, 13% of all the leucine codons should TTG, 4% of all the leucine codons should be CTA, and 13% of all the threonine codons should be ACA. The codon usage data were obtained from the Genomic Atlas Database (http://www.cbs.dtu.dk/services/GenomeAtlas/). (0.07 MB DOC) [file pone.0002394.s006.doc]

**Supporting Table S4.** Rare codons present in *E. coli* *fim* genes.

|  | **Codons present in fimbriae genes** | | | | | | | | | | | | |
| --- | --- | --- | --- | --- | --- | --- | --- | --- | --- | --- | --- | --- | --- |
| **Gene** | ***IleX, ileY* tRNA** | | ***proL* tRNA** | | ***argU* tRNA** | | | ***leuX* tRNA** | | ***leuW* tRNA** | | ***thrU* tRNA** | |
|  | **ATA**  **Expected** | **ATA**  **Present** | **CCC**  **Expected** | **CCC**  **Present** | **AGA/AGG**  **Expected** | **AGA/AGG**  **Present** | | **TTG**  **Expected** | **TTG**  **Present** | **CTA**  **Expected** | **CTA**  **Present** | **ACA**  **Expected** | **ACA**  **Present** |
| *fimZ* | 1.6 | **6** | 0.6 | **2** | 0.3/0.16 | | **2/1** | 3.1 | **4** | 0.9 | **2** | 1.9 | **3** |
| *fimB* | 1 | **6** | 0.6 | **2** | 0.7/0.3 | | **2/**0 | 2.8 | **4** | 0.8 | **2** | 1.3 | **3** |
| *fimE* | 0.8 | **3** | 0.2 | 0 | 1.1/0.5 | | **5/**0 | 2.4 | 2 | 0.7 | 0 | 1.4 | 0 |
| *fimA* | 0.4 | 0 | 0.2 | 0 | 0.1/0.06 | | **1/**0 | 1.9 | 0 | 0.6 | 0 | 2.8 | **4** |
| *fimI* | 0.7 | **2** | 1.1 | 1 | 0.4/0.2 | | **1/1** | 1.8 | **3** | 0.5 | **2** | 1.3 | 0 |
| *fimC* | 0.9 | **2** | 1.8 | **3** | 0.5/0.2 | | **1/1** | 3.4 | **7** | 1 | 1 | 2.4 | **6** |
| *fimD* | 2.4 | **4** | 4.1 | **7** | 1.8/0.9 | | **4/**0 | 10.9 | 10 | 3.3 | 2 | 8.2 | **14** |
| *fimF* | 0.5 | **2** | 1.1 | **2** | 0.2/ 0.1 | | **1/1** | 2.6 | **3** | 0.8 | **1** | 2.2 | 1 |
| *fimG* | 0.5 | **2** | 0.4 | 0 | 0.1/0.06 | | **1/1** | 1.7 | **5** | 0.5 | **1** | 2.7 | **3** |
| *fimH* | 1 | 0 | 2 | 1 | 0.3/0.1 | | **1/**0 | 2.7 | **3** | 0.8 | 0 | 3.9 | 3 |

The numbers in bold indicate more rare codons than expected according the *E. coli* codon usage for the complete genome. The expected use of rare codons in *E. coli* is as follows: 7% of all the isoleucine codons should be ATA, 13% of all the proline codons should be CCC, 4% of all the arginine codons should be AGA, 2% of all the arginine codons should be AGG, 13% of all the leucine codons should TTG, 4% of all the leucine codons should be CTA, and 13% of all the threonine codons should be ACA. The codon usage data were obtained from the Genomic Atlas Database (<http://www.cbs.dtu.dk/services/GenomeAtlas/>).
