# Supplementary material for: Protein Translation and Cell Death: The Role of Rare tRNAs in Biofilm Formation and in Activating Dormant Phage Killer Genes
Source: PLoS One. 2008 Jun 11;3(6):e2394. doi: 10.1371/journal.pone.0002394 (PMC2408971; doi:10.1371/journal.pone.0002394)
Supplement: Table S5 — E. coli strains and plasmids used. KmR, CmR, EmR, SmR and AmpR are kanamycin, chloramphenicol, erythromycin, streptomycin, and ampicillin resistance, respectively. (0.11 MB DOC) [file pone.0002394.s007.doc]

**Supporting Table S5. *E. coli s*trains and plasmids used. Km^R^, Cm^R^, Em^R^, Sm^R^ and Amp^R^ are kanamycin, chloramphenicol, erythromycin, streptomycin, and ampicillin resistance, respectively.**

| **Strains and plasmids** | Genotype/relevant characteristics | **Source** |
| --- | --- | --- |
| **Strains** |  |  |
| BW25113 | *lacI*^q^ *rrnB*_T14_ *ΔlacZ*_WJ16_ *hsdR514* Δ*araBAD*_AH33_ *ΔrhaBAD*_LD78_ | [66] |
| BW25113 *hha* | K-12 BW25113 Δ*hha* ΩKm^R^ | [66] |
| BW25113 *ybaJ* | K-12 BW25113 Δ*ybaJ* ΩKm^R^ | [66] |
| BW25113 *fimA* | K-12 BW25113 Δ*fimA* ΩKm^R^ | [66] |
| BW25113 *ihfA* | K-12 BW25113 Δ*ihfA* ΩKm^R^ | [66] |
| BW25113 *hns* | K-12 BW25113 Δ*hns* ΩKm^R^ | [66] |
| BW25113 *rzpD* | K-12 BW25113 Δ*rzpD* ΩKm^R^ | [66] |
| BW25113 *yfjZ* | K-12 BW25113 Δ*yfjZ* ΩKm^R^ | [66] |
| BW25113 *alpA* | K-12 BW25113 Δ*alpA* ΩKm^R^ | [66] |
| BW25113 *appY* | K-12 BW25113 Δ*appY* ΩKm^R^ | [66] |
| BW25113 *clpP* | K-12 BW25113 Δ*clpP* ΩKm^R^ | [66] |
| BW25113 *clpX* | K-12 BW25113 Δ*clpX* ΩKm^R^ | [66] |
| BW25113 *relE* | K-12 BW25113 Δ*relE* ΩKm^R^ | [66] |
| BW25113 *yoeB* | K-12 BW25113 Δ*yoeB* ΩKm^R^ | [66] |
| BW25113 *hha ∆Km* | K-12 BW25113 Δ*hha* ΔKm^R^ (deleted Km^R^ gene from BW25113 Δ*hha* ΩKm^R^) | This work |
| BW25113 *hha fimA* | K-12 BW25113 Δ*hha* Δ*fimA* ΩKm^R^ | This work |
| BW25113 *hha ihfA* | K-12 BW25113 Δ*hha* Δ*ihfA* ΩKm^R^ | This work |
| BW25113 *hha tqsA* | K-12 BW25113 Δ*hha* Δ*tqsA* ΩKm^R^ | This work |
| BW25113 *hha bssS* | K-12 BW25113 Δ*hha* Δ*bssS* ΩKm^R^ | This work |
| BW25113 *hha hns* | K-12 BW25113 Δ*hha* Δ*hns* ΩKm^R^ | This work |
| AG1 | *endA*1 *recA*1 *gyr*A96 *thi*-1 *relA*1 *gln*V44 *hsdR*17(r_K_^−^ m_K_^+^) | [67] |
| BL21 (DE3) | F^–^ ompT *gal* *dcm* *lon* *hsdS*_B_(r_B_^-^ m_B_^-^) λ(DE3 [*lacI lac*UV5-T7 gene 1 ind1 sam7 nin5]) | Stratagene |
| BL21 (DE3) codon plus-RIPL | *E. coli* BL21 (DE3)/pACYC-RIPL | Stratagene |
| **Plasmids** |  |  |
| pCA24N | Cm^R^; *lacI*^q^, pCA24N | [67] |
| pCA24N-*hha* | Cm^R^; *lacI*^q^, pCA24N P_T5-lac_::*hha*^+^ | [67] |
| pCA24N-*fimA* | Cm^R^; *lacI*^q^, pCA24N P_T5-lac_::*fimA*^+^ | [67] |
| pCA24N-*ihfA* | Cm^R^; *lacI*^q^, pCA24N P_T5-lac_::*ihfA*^+^ | [67] |
| pCP20 | Amp^R^ & Cm^R^; temperature-sensitive replication, thermal induction of FLP recombinase synthesis | [71] |
| pCM18 | Em^R^; pTRKL2-P_CP25_RBSII-*gfp3**-T_0_-T_1_ | [72] |
| pPROBE-*gfp*[tagless] | Km^R^; promoterless *gfp* plasmid | [37] |
| pP*fimA-gfp* | Km^R^; pPROBE P*_fimA_*::*gfp* | This work |
| pP*ihfA-gfp* | Km^R^; pPROBE P*_ihfA_*::*gfp* | This work |
| pP*ybaJ-gfp* | Km^R^; pPROBE P*_ybaJ_*::*gfp* | This work |
| pET28A-*hha* | Km^R^; *lacI*^q^, pET28A P_T7-lac_::*hha*^+^ | W. Peti |
| pBAD-*Myc*-His C | Amp^R^; *E. coli* *L*-arabinose inducible expression vector | Invitrogen |
| pBAD-*hha* | Amp^R^; P_BAD_::*hha* in pBAD-*Myc*-His C | this work |
| pBAD-*ybaJ* | Amp^R^; P_BAD_::*ybaJ* in pBAD-*Myc*-His C | this work |
| pACYC184 | Cm^R^ and Tet^R^; p15A-derived replicon; broad host range plasmid | New England Biolabs |
| pACYC-RIPL | Cm^R^ and Sm^R^; *argU*, *ileY*, *leuW*, *proL* tRNA genes under a constitutive promoter, pACYC184-derived | Stratagene |
| pVLT31 | *lacI*, Tc^R^ | [68] |
| pVLT31-*hha* | pVLT31 *plac::hha*^+^ | this work |
