# Supplementary material for: Protein Translation and Cell Death: The Role of Rare tRNAs in Biofilm Formation and in Activating Dormant Phage Killer Genes
Source: PLoS One. 2008 Jun 11;3(6):e2394. doi: 10.1371/journal.pone.0002394 (PMC2408971; doi:10.1371/journal.pone.0002394)
Supplement: Table S6 — E. coli DLP12 and CP4-57 prophage genes that are differentially regulated (P<0.05) in BW25113 biofilms upon deleting hha in LB glu after 15 h and in LB glu after 24 h. (0.04 MB DOC) [file pone.0002394.s008.doc]

**Supporting Table S6.** *E. coli* DLP12 and CP4-57 prophage genes that are differentially regulated (P < 0.05) in BW25113 biofilms upon deleting

*hha* in LB glu after 15 h and in LB glu after 24 h.

| **Gene** | **B number** | **Expression 15 h LB glu** | **Expression 24 h LB glu** | **Description** |
| --- | --- | --- | --- | --- |
| **DLP12 prophage** |  |  |  |  |
| *ybcK* | b0544 |  | -2.6 | Putative recombinase |
| *nmpC* | b0553 | -5.3 |  | Putative water filled outer membrane porin |
| *essD (ybcR)* | b0554 |  | -2.6 | Putative lysis protein S (holin) |
| *ybcW* | b0559 | -4.9 |  | Predicted protein |
| *ybcY* | b0562 | -4.9 |  | Predicted SAM-dependent methyltransferase |
| *ompT* | b0565 | -4.0 |  | Protease VII, outer membrane protein; putative porin |
| **CP4-57 prophage** |  |  |  |  |
| *alpA* | b2624 | -4.9 |  | Transcriptional activator of a P4-like cryptic prophage |
| *yfjI* | b2625 |  | -2.8 | Predicted protein |
| *yfjZ* | b2645 |  | -2.4 | Antitoxin of the YpjF-YfjZ toxin-antitoxin system |
| *ypjF* | b2646 | -6.0 |  | Toxin of the YpjF-YfjZ toxin-antitoxin system |
