# Supplementary material for: Protein Translation and Cell Death: The Role of Rare tRNAs in Biofilm Formation and in Activating Dormant Phage Killer Genes
Source: PLoS One. 2008 Jun 11;3(6):e2394. doi: 10.1371/journal.pone.0002394 (PMC2408971; doi:10.1371/journal.pone.0002394)
Supplement: Table S7 — E. coli genes induced and repressed more than 4 fold (P<0.05) in exponentially-growing suspension cells (turbidity at 600 nm of 1) upon hha overexpression in E. coli K12 BW25113/pCA24N-hha in LB medium with 2 mM IPTG at 37°C. (0.11 MB DOC) [file pone.0002394.s009.doc]

**Supporting Table S7A. *E. coli* genes induced more than 4 fold (P < 0.05) in exponentially-growing suspension cells (turbidity at 600 nm of 1) upon *hha* overexpression in *E. coli* K12 BW25113/pCA24N-*hha* in LB medium with 2 mM IPTG at 37°C.**

| **Class** | **Gene** | **B number** | **Fold change** | **Description** |
| --- | --- | --- | --- | --- |
| **HSP and chaperones** | *hha* | b0460 | 6.5 | Hemolysin expression modulating protein |
|  | *ibpB* | b3686 | 7.4 | Heat shock protein (chaperone) |
|  | *ibpA* | b3687 | 7.0 | Heat shock protein (chaperone) |
|  | *ybbN* | b0492 | 6.0 | Chaperone and weak protein oxidoreductase |
|  | *ybeZ* | b0660 | 5.0 | Putative phospHeate starvation protein induced by heat shock |
|  | *hspQ* | b0966 | 5.0 | Heat shock protein, hemimethylated DNA-binding protein |
|  | *htpX* | b1829 | 5.0 | Heat shock protein, integral membrane protein |
|  | *clpB* | b2592 | 5.0 | Heat shock induced chaperone |
|  | *htpG* | b0473 | 4.3 | Molecular chaperone, HSP90 family |
|  | *ybeY* | b0659 | 4.3 | Putative metal-dependent hydrolase induced by heat shock |
|  | *groL* | b4143 | 4.3 | Chaperone Hsp60, peptide-dependent ATPase |
|  | *hslR* | b3400 | 4.0 | Abundant heat shock protein with DNA and RNA binding |
|  | *hslU* | b3931 | 3.7 | Heat shock ATPase component of the HslVU protease |
| **Proteases** | *clpP* | b0437 | 4.3 | Heat shock preotease tHeat degrades Phd and MazE antitoxins |
|  | *clpX* | b0438 | 3.5 | ATP-dependent protease |
|  | *lon* | b0439 | 4.0 | Protease tHeat degrades misfolded proteins and antitoxins |
| **Other stress** | *cspF* | b1558 | 4.6 | Qin prophage; cold shock protein |
|  | *cspG* | b0990 | 4.3 | Cold shock protein |
|  | *proV* | b2677 | 5.0 | ATP-binding component of the transporter of glycine-betaine |
|  | *bhsA* | b1112 | 4.3 | Protein involved in stress resistance and biofilm formation |
|  | *yhgI* | b3414 | 4.3 | Protein involved in utilization of DNA as a carbon source |
|  | *emrR* | b2684 | 3.3 | Transcriptional repressor of the *emrRAB* |
| **Antitoxins** | *yefM* | b2017 | 7.0 | Antitoxin of the YoeB-YefM toxin-antitoxin pair |
|  | *dinJ* | b0226 | 4.0 | Antitoxin of YafQ-DinJ toxin-antitoxin system |
|  | *relB* | b1564 | 3.0 | Antitoxin of the RelE-RelB toxin-antitoxin system |
|  | *fxsA* | b4140 | 5.0 | Inner membrane protein tHeat provides resistance to the T7 phage |
| **Metabolism** | *frmB* | b0355 | 3.5 | *S*-formylglutathione hydrolase |
|  | *frmA* | b0356 | 8.5 | Alcohol dehydrogenase class III; formaldehyde dehydrogenase |
|  | *frmR* | b0357 | 6.0 | Repressor of *frmRAB,* induced by formaldehyde |
|  | *aceB* | b4014 | 4.6 | Malate synthase A induced by acetate |
|  | *aceA* | b4015 | 5.0 | Isocitrate lyase |
|  | *pykF* | b1676 | 4.0 | Pyruvate kinase I |
|  | *ackA* | b2296 | 3.0 | Propionate kinase/acetate kinase |
|  | *cydA* | b0733 | 4.3 | Cytochrome *bd*-I terminal oxidase subunit I |
|  | *ldhA* | *b1380* | 3.2 | Fermentative D-lactate dehydrogenase |
|  | *speD* | b0120 | 4.0 | Adenosylmethionine decarboxylase, proenzyme |
|  | *ydhD* | b1654 | 4.0 | Glutaredoxin 4 |
|  | *mrp* | b2113 | 4.0 | Putative ATPase involved in alternative pyrimidine biosynthesis |
|  | *ndh* | b1109 | 6.0 | NADH dehydrogenase II |
|  | *entC* | b0593 | 5.2 | Isochorismate synthase 1 |
|  | *nudB* | b0631 | 7.4 | Nucleoside tri-P pyrophosphohydrolase |
|  | *hisL* | b2018 | 7.4 | His operon leader peptide |
| **Cell death** | *hokD* | b1562 | 5.2 | Qin prophage; small toxic polypeptide |
| **Unknown** | *ydcY* | b1446 | 4.0 | Hypothetical protein, function unknown |
|  | *ybeX* | b0658 | 4.0 | Predicted ion transport protein |
|  | *ycgE* | b1162 | 5.0 | Predicted DNA-binding transcriptional regulator |

**Supporting Table S7B.** *E. coli* genes repressed more than -4 fold (P < 0.05) in exponentially-growing suspension cells (turbidity at 600 nm of 1) upon *hha* overexpression in *E. coli* K12 BW25113/pCA24N-*hha* in LB media with 2 mM IPTG at 37°C.

| **Class** | **Gene** | **B number** | **Fold change** | **Description** |
| --- | --- | --- | --- | --- |
| **Transport and** | *malF* | b4033 | -3.5 | Subunit of maltose ABC transporter |
| **Metabolism** | *malE* | b4034 | -5.2 | Subunit of maltose ABC transporter |
|  | *malK* | b4035 | -5.2 | Subunit of maltose ABC transporter |
|  | *lamB* | b4036 | -6.5 | Phage lambda receptor protein; maltose high-affinity receptor |
|  | *malM* | b4037 | -3.0 | Maltose regulon periplasmic protein |
|  | *malP* | c4194 | -3.2 | Maltodextrin phosphorylase |
|  | *glpT* | b2240 | -4.6 | Major *E. coli* uptake system for glycerol-3-phospHeate |
|  | *glpC* | b2243 | -4.6 | Glycerol-3-phospHeate dehydrogenase |
|  | *mglB* | b2150 | -3.0 | Galactose ABC transporter |
|  | *yhfV* | b3379 | -3.0 | Predicted hydrolase |
|  | *dctA* | b3528 | -3.0 | Dicarboxylate DAACS transporter |
| **Regulator** | *lldR* | b3604 | -3.0 | Transcriptional repressor of L-lactate utilization genes |
